# Conserved and divergent signals in 5’ splice site sequences across fungi, metazoa and plants

**DOI:** 10.1371/journal.pcbi.1011540

**Published:** 2023-10-13

**Authors:** Maximiliano S. Beckel, Bruno Kaufman, Marcelo Yanovsky, Ariel Chernomoretz

**Affiliations:** 1 Fundación Instituto Leloir, Buenos Aires, Argentina; 2 Instituto de Investigaciones Bioquímicas de Buenos Aires, Consejo Nacional de Investigaciones Científicas y Técnicas (CONICET), Buenos Aires, Argentina; 3 Departamento de Física, Facultad de Ciencias Exactas y Naturales, Universidad de Buenos Aires, Instituto de Física Interdisciplinaria y Aplicada (INFINA), Consejo Nacional de Investigaciones Científicas y Técnicas (CONICET), Buenos Aires, Argentina; University of California Riverside, UNITED STATES

## Abstract

In eukaryotic organisms the ensemble of 5’ splice site sequences reflects the balance between natural nucleotide variability and minimal molecular constraints necessary to ensure splicing fidelity. This compromise shapes the underlying statistical patterns in the composition of donor splice site sequences. The scope of this study was to mine conserved and divergent signals in the composition of 5’ splice site sequences. Because 5’ donor sequences are a major cue for proper recognition of splice sites, we reasoned that statistical regularities in their composition could reflect the biological functionality and evolutionary history associated with splicing mechanisms.

**Results**: We considered a regularized maximum entropy modeling framework to mine for non-trivial two-site correlations in donor sequence datasets corresponding to 30 different eukaryotes. For each analyzed species, we identified minimal sets of two-site coupling patterns that were able to replicate, at a given regularization level, the observed one-site and two-site frequencies in donor sequences. By performing a systematic and comparative analysis of 5’splice sites we showed that lineage information could be traced from joint di-nucleotide probabilities. We were able to identify characteristic two-site coupling patterns for plants and animals, and propose that they may echo differences in splicing regulation previously reported between these groups.

## Author summary

The main steps of the splicing process are similar across eukaryotes. However, differences in splicing factors, gene architecture, and sequence divergences suggest clade-specific features of splicing and its regulation. Using a regularized maximum entropy modeling approach we investigated two-site statistical patterns to identify conserved and divergent signals embedded in the 5’ splice sites of 30 eukaryotic species. We found that the joint dinucleotide probabilities of donor sequences carry lineage information, and we were able to trace characteristic two-site coupling patterns for plants and animals, which could reflect differences in splicing regulation previously reported between these groups. Our study also extends the observation of negative epistatic signals in mammalian donor sequences. Specifically, we showed that the average strength of coupling parameters between intronic and exonic consensus nucleotides presented destabilizing negative values, which precluded the simultaneous co-occurrence of consensus nucleotides at the intronic and exonic parts of a given donor sequence.

## Introduction

The majority of eukaryotic genes are composed of alternating stretches of DNA known as exons and introns. Introns are removed from the nascent transcript through a co-transcriptional process called splicing [1–3]. RNA splicing results from a coordinated and sequential set of biochemical reactions involving small nuclear ribonucleoproteins (snRNPs) which, together with less stably associated non-snRNP proteins, conform to the dynamic molecular machinery known as the spliceosome [1, 4]. Two types of spliceosomes are known to operate in eukaryotes: a major U2 type (with U1, U2, U4, U5, and U6 snRNPs) that processes the majority of pre-mRNAs, and a minor U12 type (with U11, U12, U4atac, U5, and U6atac snRNPs) that splices a smaller fraction of pre-mRNAs possessing so-called U12 type introns [5].

Despite some lineage-specific deviations, four major sequence cues serve to place the spliceosome at the correct locations on the immature transcripts. For the vast majority of U2 spliceosomal introns, conserved GT and AG di-nucleotides are recognized at the beginning of an intron (5’ splice site or donor site) and at its opposite end (3’ splice site or acceptor site) respectively. In addition, a branching point (BP), presenting a conserved A residue, is located 18–40 nucleotides upstream of the 3’splice site (3’ss). Finally, a poly-pyrimidine tract follows the BP and completes the necessary set of sequence cues used to guide the spliceosome assembly process [5–7].

The 5’ splice site (5’ss) is an essential element in order for splicing to take place. These donor sequences are involved in a key step in RNA splicing reactions in which the boundaries between exons and introns are recognized. At this step, U1 identifies the 5’ splice junction between adjacent exons and introns through a complex formation that depends on highly conserved base pairing between the 5’ss and the 5’ end of U1 snRNA. Specifically, the U1 snRNA forms base pairs across intron-exon junctions, potentially base-pairing with the last three positions of the exon (positions -3 to -1) and the first six positions of the intron (positions +1 to +6). This hybridization is stabilized by U1-C, one of the protein components of U1 snRNP, by establishing hydrogen bonds between its polypeptide chain and the sugar-phosphate backbone of the pre-mRNA. Because of the sequence-agnostic nature of U1-C interactions, it is particularly relevant to account for splice site sequence variability [8]. Before the first catalytic step in splicing, U1 is replaced by U5 and U6 snRNPs, for which the snRNA also needs to bind to the exonic (-3 to +1) and intronic (+5,+6) portions of the 5’ss respectively [6, 9, 10]. Artemyeva and Porter reported that U5 could also play a significant role in exon-intron boundary recognition [11]. These findings support the idea that, despite the relevance of gene context, splicing efficiency depends on 5’ss recognition to a great degree [12].

Given the relevance of donor sites, their sequence composition has been the subject of many studies aimed at uncovering non-trivial sequence patterns associated with relevant biology. Information-theoretic approaches have been widely used to provide insight into biological functionality and/or trace the evolutionary history of splicing. For instance, in the early 90s, Stephens and Schneider used information theory to analyze 1800 human splice sites. They could quantitatively estimate position-dependent contributions using a Shannon-like information measure and found that more than 80% of the sequence information (i.e. sequence conservation) was confined to the intronic part of the donor sites [13]. Sverdlov et al. also considered the position-dependent information content measure to uncover differences in the way information was distributed between exonic and intronic parts of nucleotide sequences in new (i.e. lineage-specific) and old (shared by two or more major eukaryotic lineages) introns. According to their findings, 5’ss corresponding to old introns display lower information in their exonic regions than in their intronic regions, whereas the opposite trend was seen in newer introns. This result suggests an evolutionary splice signal migration from exons to introns during evolution [14]. More recently, Iwata and Gotoh performed a comparative analysis of 61 eukaryotic species. Using annotated splice sites from transcriptomic data, they showed that although donor site motifs resembled each other (suggesting that the spliceosome machinery is well conserved among eukarya) they exhibited some degree of specificity [15, 16].

Single-site statistics, such as logos or consensus sequences, do not fully characterize the complex statistics embedded in 5’ss sequences. There have also been attempts to infer higher correlation patterns in donor site sequences. One such result was reported by Stephens and Schneider’s research. Using information theory they were able to find significant mutual information values of approximately 0.05, 0.07 and 0.04 bits, between human 5’ss positions (-2,+4), (-1,+5) and (-2,+5) respectively [13]. Almost ten years later, following a completely different route, Thanaraj and Robinson used decision trees to predict exon boundaries and found that long-range dinucleotide associations (-1,+5) and (-2,+5) carried significant splicing signals [17]. Couplings between the (-1,+5) and (-2,+5) position pairs have also been featured in a comparative human-mouse genomic study carried out by Carmel et al. [18]. Sahashi et al. also analyzed two-site correlations in human 5’ss and reported that non-complementary nucleotides to U1 snRNA at specific positions were compensated by complementary nucleotides at other positions, suggesting that a stretch of complementary nucleotides in either an exonic or intronic region is essential for proper splicing [19]. Denisov et al. presented evidence to support and extend this idea. Through a comparative analysis of the genomes of three mammals they found a well-defined pattern of epistatic interactions between nucleotides occupying different positions along the donor site. While the strength of correlations within both the intronic and exonic sections was found to be positive (i.e. positive epistatsis), nucleotide strength correlations between said regions were found to present negative epistasis [20]. Another relevant study on RNA motifs in connection with splicing signals was presented by Yeo and Burge [21]. Considering an entropy maximization framework, they showed that maximum entropy distributions consistent with different sets of constraints (i.e. enforcing low-order marginal probability distributions to match empirical values) could be used to recognize true RNA splice sites in primary transcript sequences. They proposed a likelihood ratio statistic to discriminate real splice sites from decoys and showed that relevant constraints could be identified by studying the amount of entropy reduction induced by their procedure [21].

In decent decades, models rooted in the principle of maximum entropy have been widely applied to various biological problems [22, 23]. The success of this modeling strategy is based on the observation that low-order correlations, primarily pairwise interactions, play a significant role in many biological systems. Consequently, accurate approximations of joint probability distributions for systems with multiple interacting elements can often be achieved by considering only pairwise interactions among the components of the system. This approach has been applied to study biological systems of very different levels of organization, including protein sequences [24–26], networks of real neurons [27, 28], and bird flocking [29, 30]. The present study aims to delve further into donor sequence regularities by following a similar route. Considering a maximum entropy framework, we developed a generative probabilistic model allowing an analysis of sequence composition for donor sequences in several eukaryotic species.

Our approach sought to focus on lineage specific signatures in order to gain biological insights about splicing; however, in contrast to Gotoh’s work [15], we explicitly considered two-site marginal probabilities for donor sequences. To the best of our knowledge, this is the first study to demonstrate that phylogenetic signals are embedded in two-site correlation patterns. Our work is based on the analysis of two-site marginal probabilities for donor sequences of 30 eukaryotic species, significantly expanding the number of taxa analyzed compared to previous studies that examined two-site epistatic interactions in 5’ss. For example, Schneider and Thanaraj analyzed only human sequences [13, 17]. Additionally, Sverdlov, Carmel, and Denisov conducted comparative studies of eight eukaryotic genomes [14], human and mouse genomes [18], and three mammal genomes [31], respectively.

The remainder of this manuscript is organized as follows. First, we introduce and explain the design of the modeling framework. We then discuss how our regularized models effectively captured the observed one-site and two-site nucleotide frequencies. In addition, we demonstrate how our approach progressively disentangles the hierarchy of coupling parameters while maintaining the ability to reproduce observed correlations at a desired level of precision. Next, we examine various aspects of the data-driven energy distribution function that naturally emerges from our maximum entropy approach. Subsequently, our focus shifted towards characterizing the identified coupling patterns. We conducted a comparative analysis of these patterns to identify robust and conserved signatures, shedding further light on previously detected epistatic signals within the donor sites. We also explored the structural characteristics of di-nucleotide two-site probabilities, which encode phylogenetic information and enable the characterization of plant-specific and animal-specific patterns. Finally, in the last section of this paper, we discuss the biologically relevant implications of our findings and present our conclusions.

## Materials and methods

### Analyzed genomes

Our analysis used genomic data from 30 eukaryotic species (Table A in S2 Text), including five fungi, eight plantae, and 17 metazoan genomes, with the aim of uncovering specific features for these groups. We considered nine-nucleotide (9-nt) long sequences to analyze donor sites, in accordance with previous studies that found that most relevant information content was restricted to the region including the last three nucleotides of the exon and the first six intronic positions [13, 15, 19, 20, 32]. We wrote a custom script to automatically extract 9-base length donor sequences based on the genome sequences (FASTA files) and annotations (GTF/GFF3 files) downloaded from Ensembl.

### Statistical model

For each species, our aim was to estimate the joint probability distribution function, $P(\vec{S})$, associated with an observed ensemble of 5′ss. Each ensemble was a set of 9-nt long sequences (the last three exonic positions followed by the first six intronic positions), expressed as $\vec{S}=\left(s_{-3},s_{-2},s_{-1},s_{1},s_{2},s_{3},s_{4},s_{5},s_{6}\right)$ where *s*_*i*_ ∈ {*A*, *C*, *G*, *T*}. Under the hypothesis that the sought distribution should be compatible with observed 1-site and 2-site marginal probabilities *f*_*i*_(*s*_*i*_) and *f*_*ij*_(*s*_*i*_, *s*_*j*_), we implemented an entropy maximization approach to find the minimal structured distribution consistent with these constraints, A brief introduction to maximum entropy models was included in S1 Text and further technical details can be found in [23, 33]. Under this framework the estimated probability distribution function $\hat{P}\left(\vec{S}\right)$ takes the form of Boltzmann distribution:

$$\begin{array}{c} \hat{P}\left(\vec{S}\right)=\frac{1}{Z}e^{-E_{d}\left(\vec{S}\right)} \end{array}$$ (1)

where

$$\begin{array}{c} E_{d}\left(\vec{S}\right)=-\sum_{i=1}^{9}h_{i}\left(s_{i}\right)-\sum_{i<j}^{9}J_{ij}\left(s_{i},s_{j}\right) \end{array}$$ (2)

plays the role of a data-driven energy. The partition function, $Z=\sum_{\vec{S}}e^{-E_{d}\left(\vec{S}\right)}$, is simply a normalization constant. Variables *h*_*i*_(*s*_*i*_) and *J*_*ij*_(*s*_*i*_, *s*_*j*_) are the fitting parameters of our model. Overall, this model contains 36 single-site parameters *h*_*i*_(*s*_*i*_) (four possible bases in nine sites) and 576 two-site interactions *J*_*ij*_(*s*_*i*_, *s*_*j*_) (16 base combinations for 36 site-pairs) to be estimated. For convenience, our notation will hence omit the explicit dependency on *s*_*i*_ variables (e.g. *J*_*ij*_(*s*_*i*_, *s*_*j*_) ≡ *J*_*ij*_).

### Time-tree

To make use of phylogenetic data, we obtained a time-tree from a publicly available database at timetree.org (last access December 20, 2021) [34]. Two of our 30 analyzed species were not found in the said database and were replaced by evolutionarily close species for the sake of phylogenetic calculations. Specifically, Magnaporthe oryzae (mor) was replaced by Pseudohalonectria lignicola (both from the Magnaporthacease family) and Coprinopsis cinerea (cci) was replaced by Coprinopsis lagopus (both from the genus Coprinopsis).

### Phyogenetic signals

We used the Maddison-Slatkin randomization procedure [35] to survey statistically supported associations between 41 non-zero model coupling parameters and phylogenetic signals. This non-parametric bootstrapping approach is employed to generate a distribution of expected values for a given test statistic. Our chosen test statistic was a parsimony score defined as the number of changes (in parsimony steps) of the binary trait of interest, that is, the presence/absence state of the analyzed coupling parameter. Specifically, for each coupling parameter a presence/absence binary state was assigned to taxa 10000 times and parsimony scores (Sankoff methodology [36]) were estimated for each random assignment using the function *parsimony* implemented in the *phagorn* package for the R programming language [37]. Bonferroni-corrected p-values were estimated from the fraction of random events with parsimony scores equal to or greater than the observed value.

### Dendrograms inferred from two-site probabilities

As a distance function, we considered the Euclidean metric between the vectorized upper-triangular matrices of two-site probabilities *P*_*ij*_ generated by the models fitted to the analyzed species. A dendrogram was then constructed using the complete agglomeration method.

### Dendrogram comparisons

The functionality implemented in the R-package *dendextend* [38] was used to carry out dendrogram comparisons. We found *tanglegrams*, a specific type of diagram to visually compare two dendrograms, to be an informative tool when used to qualitatively compare pairs of hierarchical ordinations. In addition, the function *Bk-permutations* was used to carry out a bootstrap analysis (10000 permutations) of Fowlkes-Mallows indices [39] to compare partitions induced at different cut-levels from the dendrograms of interest (see Section G in S1 Text).

## Results

### Maximum entropy model for 5’ss

**Fig 1:**
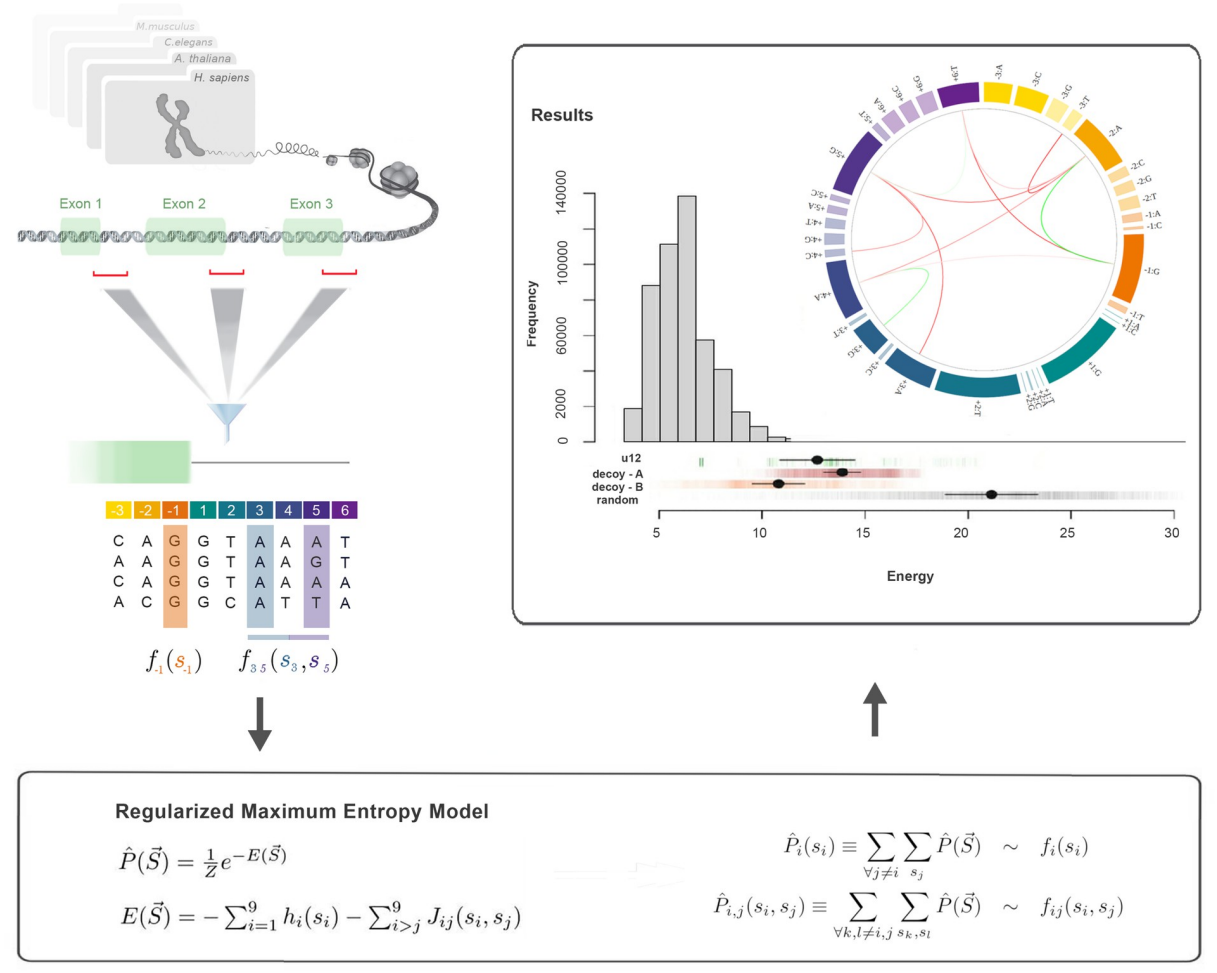
The left panel of the figure shows the workflow diagram of our modeling methodology: we identified 9-base length annotated donor sequences from each analyzed genome and estimated 1-site and 2-site marginal probabilities *f*_*i*_(*s*_*i*_) and *f*_*ij*_(*s*_*i*_, *s*_*j*_). The bottom panel displays mathematical expressions defining the maximum entropy model as a function of the fitting parameters *h*_*i*_(*s*_*i*_) and *J*_*ij*_(*s*_*i*_, *s*_*j*_). In the upper-right panel, we present the energetics of human donor sequences (*γ* = **0.025**), showing the distribution of data-driven energy values for the 502197 5’ss sequences observed from the human genome. Rows are added to represent the distribution of u12 junctions along with decoy-A, decoy-B, and random sequences (sample of 10000), denoted by green, red, orange, and gray ticks, respectively. Black points represent mean values, whereas black lines represent *σ* energy intervals. The inset shows a circos representation of coupling interactions, where an outer ring of 36 boxes represents single-site relative frequencies. Warm colors are used for the three exonic sites, while cold colors represent intronic sites. The area of each box is proportional to the nucleotide-site observed probability *f*_*i*_(*s*_*i*_). Positive and negative couplings are depicted connecting different site-base combinations in green or red curves, respectively.

The first step in our procedure was to use a maximum-entropy framework to fit the discussed parameters to the observed sequence ensembles. The workflow for a single given species is illustrated in Fig 1. After identifying the ensemble of donor sequences, we estimated the relative nucleotide appearance frequencies at each single site *f*_*i*_(*s*_*i*_), as well as two-site frequencies between bases *f*_*ij*_(*s*_*i*_, *s*_*j*_). These experimental measurements were then used to infer the fitting parameters *h*_*i*_(*s*_*i*_) and *J*_*ij*_(*s*_*i*_, *s*_*j*_) of our model $\hat{P}\left(\vec{S}\right)$ (see Methods section for details).

#### Statistical coupling patterns

The inset of the right panel of Fig 1 shows a circos graphical representation of the coupling connectivity pattern obtained for human 5’ss using the *γ* = 0.025 model. The outer ring of 36 boxes represents the relative frequencies of the four possible nucleotides occurring in each position in different 9-site donor sequences. Warm colors were used for the three exonic sites, whereas cold colors represented the six intronic sites. Four blocks of different areas, representing the frequency of a given base *A*, *C*, *G*, *T*, were included for each site (i.e. the largest area was associated with the site’s consensus base). Positive and negative couplings are depicted by green and red curves, respectively. We noticed negative interactions between exonic and intronic consensus site-base combinations. We also recognized positive stabilizing interactions between consensus pairs within exons and introns. A detailed analysis of these patterns is presented in the following sections.

#### Data-driven energetics

According to the estimator of sequence probabilities introduced in Eq 1, the energy function defined in Eq 2 provides a quantitative measure of how often a given sequence can be found along the entire genome. Low energy sequences correspond to the most prevalent 5’ss, whereas high-energy sequences are associated with rare and infrequent donor sequences.

The right panel of Fig 1 shows the frequency distribution of donor sequence energies for the 502197 exon-intron annotated boundaries in the human genome (*γ* = 0.025 model). We can observe a slight skew in the energy distribution, toward a high energy value. Ninety percent of the sequences had data-driven energy values laying in the *E*_*d*_ ∈ [4.1, 9.7] energy range. On the low-energy side, the sequence of minimal energy, ${\vec{S}}^{*}=\left\{C,A,G,G,T,A,A,G,T\right\}$, exhibited perfect complementarity to the U1 snRNA stretch, presenting an energy value of $E_{d}\left({\vec{S}}^{*}\right)=3.50$. This particular state was found to be the global minimum in the data-driven energy landscape of the entire ensemble of natural sequences (see Section F in S1 Text).

Although the main goal of this study was not to identify splice sites, valuable insights can be obtained by analyzing the results obtained when applying data-driven energetics to four different kinds of sequences not associated with splicing. We began by considering an ensemble of 10000 randomly generated sequencies as a null reference dataset for the data-driven energy scale (row of gray ticks in Fig 1). This null-model distribution can be characterized by its mean energy and standard deviation *E_random_* = 21.14.6, and serves to establish a proxy for the completely disordered limit. Another relevant ensemble is depicted by green ticks in Fig 1; this corresponds to 136 5’ss reported to be targets of the minor U12 spliceosome according to the Intron Annotation and Orthology Database [40]. A high energy bias can be observed for these sequences, suggesting that, from our model’s point of view, these U12 donor sites involved rather unusual sequences that came from a different statistical distribution. We verified that our main findings, such as coupling patterns, remained unaltered regardless of whether these sequences were present or absent from our training dataset. The final analysis involved the creation of a “decoy sequence” dataset. We randomly sampled 100,000 GT loci from the *Homo sapiens* genome. For each selected locus, we retained 3-nt upstream and 4-nt downstream of the GT dinucleotide, resulting in a 9-nt decoy sequence. Given the fact that there are approximately 500,000 annotated 5’ss sequences in the human genome and about 150 million GT dinucleotide sites, the probability of randomly sampling a 5’ss locus is approximately 0.3%. The decoy ensemble was comprised of 12,996 unique sequences. Among them, approximately 60% (7,629) were not found in the set of 5’ss sequences, whereas the remaining 40% of non-donor sequences (5,367) matched with at least one annotated 5’ss sequence. We refer to these disjoint groups as decoy-A and decoy-B sequence sets, respectively. In the right panel of Fig 1 decoy-A and decoy-B sequences are sown in rows of red and orange ticks, respectively. Decoy-A sequences (centered at 〈*E*_*A*_〉 = 13.9) presented the highest energy values of both decoy groups, emphasizing the importance of the nucleotide composition surrounding the GT di-nucleotide. The decoy-B set (centered at 〈*E*〉 = 10.8) presented significantly lower energy values (with a p-value of 2.2e-16 on a Wilcoxon test) but still lay on the high energy tail of the U2 sequences. Although the sequence composition of the decoy-B loci was identical to that of the identified donor sites, they were not annotated as splice sites in the genome. This discrepancy may be attributed to either an incomplete genome annotation or the presence of unfavorable genomic contexts that hinder the effective recognition of these loci by the spliceosome machinery.

Finally, Fig 1 suggests that the data-driven energy (Eq 2) provides a reasonable scale for the characterization of donor sites, going from completely ordered (perfect match against U1) to completely disordered sequences. The vast majority of exon-intron boundary sequences lies between these extreme behaviors, in a region of natural variability where recognition is possible, but full binding with the recognition machinery is avoided. In addition, we found that our data-driven characterization correlated with the energy scales calculated using physics-based methodologies. Not only did the perfect complementary sequence to U1 (i.e. the one that minimized the binding energy) present the minimal energy value but, more generally, we can observe a monotonically increasing relationship between our modelled sequence energies and estimations of the biochemical dimerization energy of 5’ss sequences against the U1 RNA stretch (see Fig A in S2 Text).

#### Transcriptomic vs Genomic 5’ss models

The dataset used for model training plays a critical role in statistical learning. In our case, we aimed to assess whether the genomic or transcriptomic origin of the data could have possibly biased our results.

**Fig 2:**
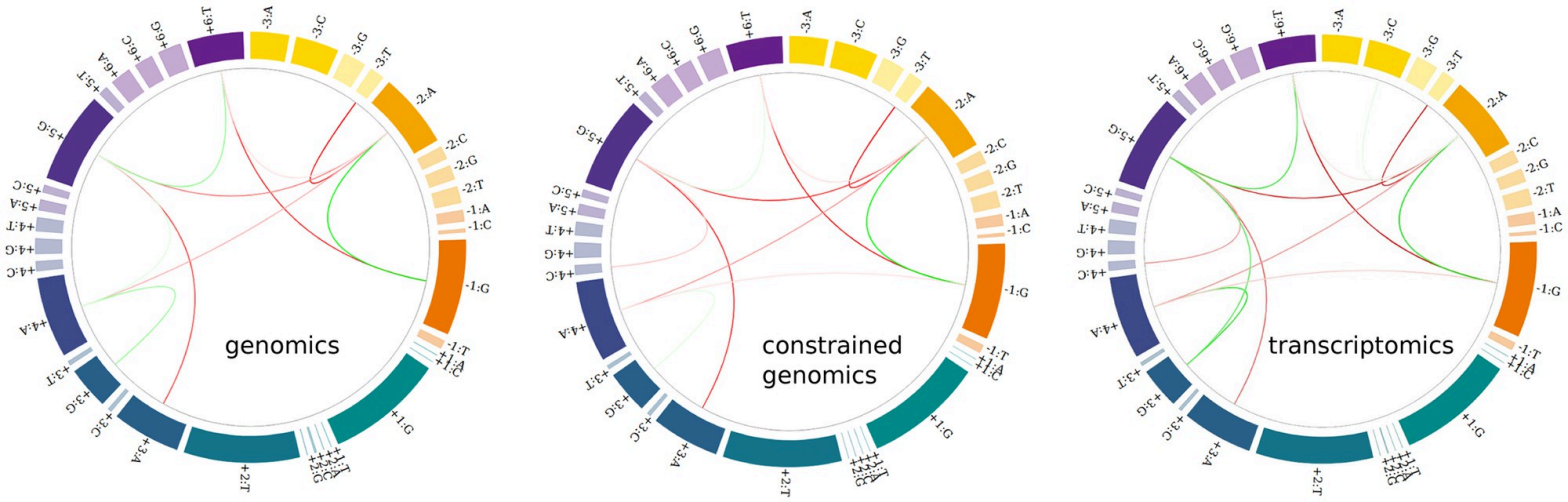
Circos diagrams illustrating coupling patterns for complete (genomics) and GT-restricted (constrained genomics) sets of human annotated donor sequences are shown in left and center panels. Interactions inferred from junctions transcriptionally expressed in normal human samples according to RJunBase (transcriptomics) are shown in the right panel.

We first fitted two different *γ* = 0.025 models considering the complete (502497) and GT-restricted (488939) ensemble of human donor sequences extracted from genomic annotations. We additionally trained a model supported by transcriptomic data obtained from the publically available resource RJunBase, located at http://www.rjunbase.org/. This database integrates information about RNA splice linear junctions in normal and cancerous human tissues present in 10,283 RNA-seq samples from The Cancer Genome Atlas (TCGA) and from the Genotype-Tissue Expression (GTEx) portal [41]. We kept only annotated linear junctions of protein-coding genes with a median expression level greater than five in normal tissues (114745 5’ss, aprox. top 50%). Fig 2 shows a circos representation of the obtained coupling patterns.

It can be seen from this figure that the regularized models were able to learn very similar coupling patterns from these four ensembles, supporting the robustness of the inferred pairwise connectivity patterns.

### Conserved coupling patterns

We identified conserved signatures by examining the average strength of coupling parameters found between consensus (C) and non-consensus (NC) bases at the exonic (E) and intronic (I) parts of donor sequences. Looking at those values obtained with *γ* = 0.025 models (Table B in S2 Text), we found that interactions between consensus bases, either at intronic (IC-IC mean interaction) or exonic sites (EC-EC mean interaction) consistently presented stabilizing positive values. Similarly, we found a consistent bias toward negative interaction values between intronic and exonic consensus sites (IC-EC) for almost all analyzed species. These results were also observed for less regularized (and hence more complex) *γ* = 0.015 models (see Table C in S2 Text) and suggest a statistical aversion to the co-appearance of consensus bases at intronic and exonic sites simultaneously. This observation agrees with (and extends) already presented evidence of negative epistatic signals found in mammalians donor sequences [19, 20].

### Divergent coupling signals

Despite the high degree of conservation of the spliceosome, single site frequencies have been shown to display a non-trivial degree of specificity in eukaryotes [15, 42]. Notably, we found that the two-site marginal probabilities captured by our model also exhibited this behavior. In fact, the hierarchical structure obtained from *P*_*ij*_ probabilities is identical to that inferred from logo motifs, that is, one-site statistics *P*_*i*_ (see Fig B in S2 Text).

**Fig 3:**
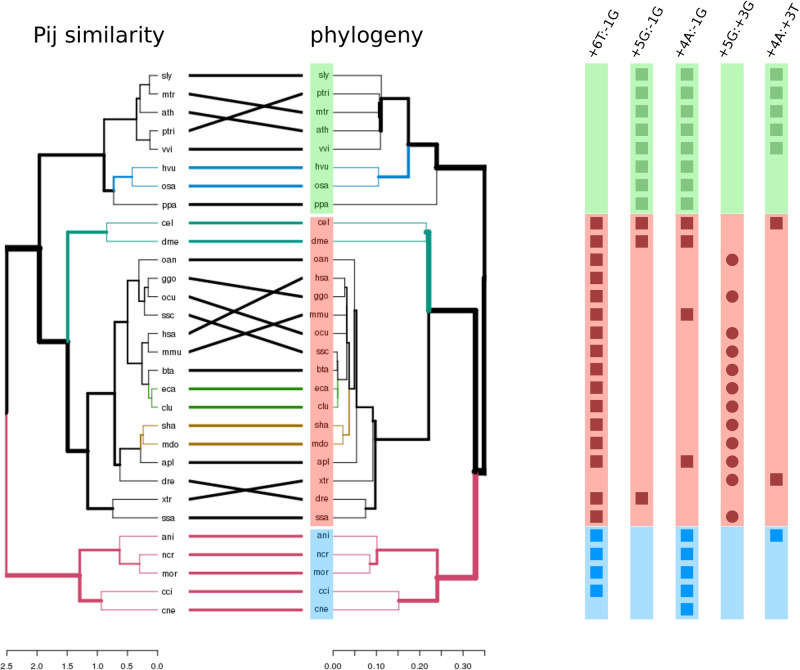
Divergent coupling patterns. **Left**, *P*_*ij*_-induced dendrogram and phylogenetic tree laid out as a tanglegram. The x scales correspond to Euclidean distances between vectorized *P*_*ij*_ values and the timetree’s divergence time estimations, respectively. Green, red and blue are used to highlight plant, animal and fungal species respectively. **Right**, presence/absence matrix for five modelled interaction parameters identified as statistically significant (see text). Square and circle shapes were used for negative and positive interactions respectively.

To compare the hierarchical structure of *P*_*ij*_ probabilities against pre-existing evolutionary data, we turn to the tanglegram shown in the left panel of Fig 3. This plot contains two dendrograms (with the same set of labels), one facing the other, with their labels connected by lines. The left-most dendrogram was constructed from two-site probabilities *P*_*ij*_ for *γ* = 0.025 models, whereas its counterpart to the right is the aforementioned time-tree inferred from phylogenetic signals (see Fig C in S2 Text). We can observe from the figure that the structure that emerges by comparing two-site statistics *P*_*ij*_ displayed strong concordance with the underlying phylogenetic relationships between the species compared. In particular, we observed a clear separation between plants, animals and fungi. Several quantitative figures of merit support this hypothesis. For instance, a cophenetic correlation value of 0.9 was found between both dendrograms, along with statistically significant Fowlkes-Mallows indices (*p*_*v*_ < 10^−4^) for almost the entire range of k-cluster groups (2 < *k* < 29) (see Fig J in S1 Text). Additionally, the entanglement value of 0.01 suggested a high quality tanglegram layout (see Materials and methods).

### Coupling parameters and phylogenetic signals

The left panel of Fig 3 suggests that the two-site statistics captured by our model contain information that is compatible with phylogenetic signals. Thus, we may wonder whether this signal is also reflected in a given subset of the coupling parameters found by our maximum entropy model.

For each non-zero *J*_*ij*_ identified in any of the analyzed species, we performed a Maddison-Slatkin procedure as a test for a phylogenetic signal (see Materials and methods). We found five interactions displaying statistically significant association with the said signal, and report the corresponding results in Table 1. The number of observed evolutionary steps for the analyzed coupling parameter, inferred using the Sankoff parsimony methodology [43], is shown in column MS.obs. The minimum, median and maximum number of evolutionary steps detected in 10000 bootstrapped samples, and Bonferroni adjusted p-values are reported in columns MS.null and MS.pv respectively. The corresponding presence/absence of these interactions in different species is shown in the right panel of Fig 3. For clarity, we use a simplified notation for the coupling parameters: for instance, -1G:+6T denotes the *J*_−1,+6_(*G*, *T*) parameter.

**Table 1:** Phylogenetic signals associated with coupling parameters. Each row depicts a model parameter found to be statistically significant in discerning animals, plants and/or fungi by a Maddison-Slatkin test. For each coupling parameter (first column) we can see the number of plants, animals and fungi the interaction was detected in (second, third and fourth columns). The observed number of Sankoff inferred evolutionary steps is reported in the fifth column. Minimum, median and maximum values for this quantity for bootstrapped samples are reported in the sixth column (comma separated values). Bonferroni corrected p-values were included in the last column of the table.

Parameter	plants	animals	fungi	MS.obs	MS.null	MS.pvD
-1G:+6T	0	16	4	3	3,9,10	4.1e-3
-1G:+5G	8	3	0	3	4,9,11	0
-1G:+4A	8	4	5	3	3,10,13	4.1e-3
+3G:+5G	0	12	0	4	4,10,12	4.1e-3
+3T:+4A	5	2	1	3	3,7,7	5.0e-2

As expected, we can see from this figure that the coupling patterns identified as significant were selectively present in plants, animals, or fungi. The first three entries in Table 1 correspond to negative interactions between intronic and exonic consensus occurrences; specifically, those connecting the -1G exonic site-base combination with the last three intronic positions. The interaction -1G:+6T was found to be a trait for animals and fungi, whereas -1G:+5G was mainly present in plants. On the other hand, the -1G:+4A coupling could be considered a shared trait in plants and fungi.

The fourth entry in Table 1 corresponds to a positive intron-intron interaction between consensus nucleotides (+3G:+5G), which appeared in 70% of the analyzed metazoans and was completely absent in plants and fungi. Finally, +3T:+4A negative coupling was found in 60% of the analyzed plant genomes.

Our findings suggest that non-trivial phylogenetic information is present in two-site correlations, which were used by our model to infer statistical coupling patterns. The strongest phylogenetic signals were reported for coupling parameters involving a negative interaction between a consensus nucleotide in the last exonic position and consensus nucleotides located at the last three intronic positions.

### Consolidated models

**Fig 4:**
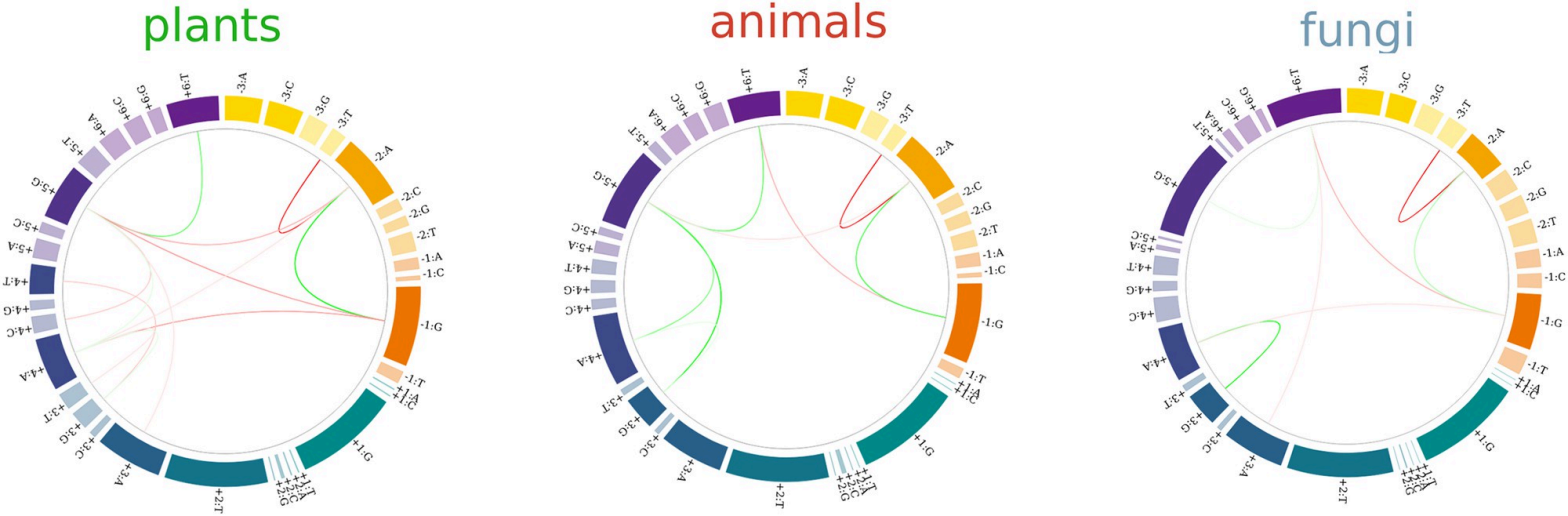
Pairwise interaction patterns. Circos diagrams for coupling parameters (*γ* = 0.025 model) identified for plants, animals and fungi donor sequences.

To further investigate the specificity of coupling signatures, we examined whether distinct coupling patterns could emerge by grouping species into animals, plants, and fungi categories. To accomplish this, we analyzed a single consolidated ensemble of sequences per group. These consolidated ensembles were constructed by uniformly and proportionally sampling 800000 splicing sequences from 17 metazoan genomes to create an ‘animal’ ensemble, 800000 splicing sequences from eight plant genomes to form a general plant ensemble, and 161547 sequences from five fungal genomes to establish a fungus ensemble. The smaller size of the fungal dataset was due to the limited number of donor sites in the smaller fungal genomes. We then fitted the maximum entropy models for each dataset to obtain the representative coupling patterns for each analyzed group. The resulting coupling diagrams for the *γ* = 0.025 models are shown in Fig 4. This regularization value was sufficiently strict to highlight the main coupling patterns corresponding to the most significant two-site interactions obtained from each dataset (see Fig C inset in S1 Text).

We can observe the re-emergence of coupling parameters identified as important in the previous sections. Regarding conserved signals, positive couplings between consensus sites within the intronic and exonic sections of the splicing junction (e.g. -2A:-1G or +5G:+6G) were present in plants, animals and fungi. Another ubiquitous interaction was the negative coupling between non-consensus and consensus exonic nucleotides -2A:-3T.

We may also notice patterns consistent with the divergent behavior uncovered by the Maddison-Slatkin analysis in the last section. A strong negative coupling was observed between +6T and -1G occurrences in the metazoan and fungal groups, which was replaced by a negative interaction between +5G and -1G occurrences in the plant group. The negative coupling -1G:+4A was detected in plants and fungi, but not in metazoans, whereas the positive coupling +3G:+5G was a signature exclusively detected in this last group. These patterns persisted when regularization levels were relaxed, such as in the *γ* = 0.015 case shown in the second row of Fig D in S2 Text.

## Discussion

The molecular recognition of 5’ss faces numerous challenges. First, with the exception of the first GT intronic nucleotides, natural 5’ss sequences are highly degenerate. Second, recognition occurs at different stages of the splicing cycle from various complexes that bind completely or partially to the 5’ss sequence. Third, not only are snRNA complexes involved in recognition, but many proteins present within them are also important for binding stabilization. Finally, splicing occurs in a genomic context in which factors such as gene structure and the presence of cis and/or trans signals can significantly influence the fine regulation of each splice site. In this study we aimed to mine conserved and divergent signals in 5’ donor sequences. The rationale for our approach was that the underlying statistical patterns in donor sequences composition should reflect aspects of the complex scenario described.

Our entropy maximization strategy allowed us to recapitulate previous results within a unified framework and to gain new insights into the regularities embedded in the statistics behind donor splicing sequences. For instance, the data-driven energy scale *E*_*d*_ (Eq 2) naturally accommodates the idea behind the SD-Score (defined as the logarithm of the frequency of a donor sequence) introduced by Sahashi et al. to predict splicing outcomes observed in artificially designed minigenes [19]. *E*_*d*_ also correlated with the estimated dimerization energies against U1 snRNA (see Fig A in S2 Text). Despite the large number of cis and trans elements that contribute to the regulation of splicing, this finding suggests that *E*_*d*_ by itself might, to some degree, reflect the strength of a given donor site. In this sense, *E*_*d*_ not only serves, by definition, to quantify the extent to which a given sequence is represented along a genome, it also provides a meaningful scale for measuring the degree of complementarity to the spliceosome machinery. The majority of naturally occurring sequences presented intermediate *E*_*d*_ values (Fig 1), suggesting a statistical aversion to 9-nt perfect matches. This concurs with previous observations stating that a minimum of 5–6 Watson-Crick pairs are required for splice site recognition, but excessive pairing (>7 bases) is detrimental [18]. This loose binding could favor splicing reaction processivity, and favor scenarios in which effective binding is regulated by third parties.

With the aid of our model we also identified general two-site interaction patterns, which suggests that this free-energy deficit is rooted in a non-trivial spatial distribution of matching pairs along splice site sequences. Despite some degree of specificity, couplings between consensus sites within exonic and intronic parts of donor sequences were biased toward positive values (columns 5 and 8 of Table B in S2 Text). In contrast, predominantly negative interactions were found between consensus nucleotides lying on different sides of the exon-intron boundary (first column of Table B in S2 Text). These results are in accordance with previous observations obtained from human and mouse donor sequence data [18–20] and support the idea that a high complementarity level is alternatively favored at either the exonic or intronic sections of the donor splice site.

Consistent identification of two-site couplings suggests that nucleotide occurrences at different positions of the 5’ss are not independent. While this observation has been reported previously, we were able to show that the joint probabilities of nucleotide pairs carry biologically meaningful information. This is shown through dendrograms constructed from the probability distributions, which closely follow the phylogenetic relationships between the analyzed species (see Fig 3). Donor splice site sequences constitute recognition sites not only for U1 snRNA in the early spliceosome complex, but also for U6 and U5 in the pre-catalytic reaction step. Subtle compensation mechanisms could then be expected to occur to enssure splicing fidelity even in the presence of large site variability. In this conext, our findings suggest uncovered statistical regularities to echo divergent evolutionary processes linked to structural specificities in the splicing machinery used to define exon-intron boundaries. However, establishing a direct relationship between the interactions identified by our model and the details of the splicing recognition mechanism can be a challenging task to accomplish. Studies such as Schwartz et al. [42] have not been able to definitively show a clear correlation between sequence variations generated at 5’ss sites and the complementary sequence in the U1 snRNA. This is partly because splice site recognition results from a large number of factors. For example, a recent study [44] determined that the fidelity of the spliceosome is highly influenced by a large number of accessory factors that are not indispensable for the splicing process to take place.

Our results also suggest that phylogenetic signals can be captured by coupling parameters within our model. These associations proved robust insofar as they were detected for both strongly and weakly regularized models, when coupling patterns were analyzed on a species-by-species basis, and when donor sequences were consolidated into separate groups for plants, animals and fungi.

Many of the two-site interactions detected in this contribution for different species have already been reported in the literature, in the context of narrower studies focused on human, mouse or other mammalian genomes [13, 17–21]. For instance, our model readily captured positive couplings between intronic +4:+5 and +5:+6 position pairs, as well as a negative -2:+5 interaction. Notably, we found a -1:+5 negative coupling for plants; however, unlike the results obtained in previous studies on humans and mammals, we did not detect it for metazoans. Both sites linked by this interaction are highly informative (see Fig E in S2 Text) and previous studies reporting a negative interaction between them includes the work of Yeo and Burge, who analyzed 12700 introns of 1821 non-redundant transcripts [21], and the contribution of Carmel and collaborators, who inferred the -1G:+5G compensatory relationship from a comparative analysis of 8869 human-mouse homolog exons [18]. Notably, a different result was reported in a recent study by Wong et al., who quantified the activity of 32,768 unique artificially engineered 5’ss sequences in three different genomic contexts and found a positive epistatic interaction between these sites [12]. While our comparative analysis managed to detect this interaction in a few animals, *Homo sapiens* was not one such species; neither did our model retrieve the said coupling parameter when trained with a consolidated metazoan ensemble. This is supported by the low observed correlation between the -1G and +5G occurrences in our metazoan 5’ss dataset, indicating that this discrepancy comes from the data, and not from model training. This result was robust, whether we made use of the complete set of annotated 5’ss, or the calculation was restricted to donor sequences presenting the canonical GT nucleotides at the start of the intron section (see Fig F in S2 Text). Moreover, we found no major qualitative differences in the coupling patterns inferred either from GT-5’ss or from the complete set of annotated donor sequences (see Fig G in S2 Text).

According to our model, the compensatory behavior reported between the last exonic and intronic sites can be understood through an interwoven set of stabilizing (+5G:+6T and -1G:-2A) and destabilizing (-2A:+5G and -1G:+6T) interactions (see Fig 4). This complex interaction pattern is consistent with recent observations by Artemyeva and Porter, who reported that the base pair composition at positions -1 and -2 was significantly altered based on the occurrence (or lack thereof) of a +5G nucleotide (Fig 4 in [11]). In addition, the relevance of position +6 has already been noticed by Carmel et al. in connection with splicing aberrations leading to familial dysautonomia [18]. This study provides experimental evidence that a base pair at position -1 prevents the aberrant splicing of the 20th intron of the IKBKAP gene caused by a mispair of position +6 with U1 snRNA. We found a similar compensatory setup between intronic (+5 and +6) and exonic (-2 and -1) positions in plants and animals. In the case of plants, however, we detected a -1G:+5G negative coupling which replaced the -1G:+6T interaction observed in metazoans (see Fig 4). Notably, these two-site interactions affected intronic positions that had fairly low information content in plants (0.27 bits and 0.21 bits for positions +5 and +6 respectively, see Fig E in S2 Text) suggesting that the detected network of pair-wise interactions could play a major role in the way these species deal with sequence variability.

Several differences have been highlighted between plants and animals in terms of splicing mechanisms. Arguably the most straightforward differences are the large differences reported in typical intron lengths and the prevalence of different types of splicing events: exon skipping in animals and intron retention in plants [5, 45]. Many recent studies have pointed out not only gene-architecture but also functional differences between alternative splicing in animals and plants [46–49]. For instance, although the spliceosome in plants has not yet been isolated, the number of splicing factors identified in *Arabidopsis thaliana* nearly doubles taht observed in humans [50]. In addition, the prevalence of intron retention events coupled with NMD transcript degradation and nuclear sequestration suggests that, in contrast to animal organisms, splicing in plants could play a major functional regulatory role, closely related to stress response, as well as expanding proteomic diversity [5, 45, 51–53]. In this context, our results highlight significant differences in two-site interactions involving donor site nucleotide positions relevant to functional and evolutionary considerations. Based on our findings, we believe that further investigation is warranted concerning the connection between these differences and the distinctive mechanistic features of the splicing processes in plant and animal species.

## Conclusion

In this study, we employed a maximum-entropy approach to obtain regularized probabilistic generative models of donor sequences for 30 different eukaryotic species.

Our model incorporates a data-driven energy scale that captures the abundance of a given sequence within a specific genome. This energy statistic serves as a practical measure for characterizing sequences, representing the continuum between completely ordered and disordered sequence states.

We also showed that the joint di-nucleotide probabilities in donor sequences carry lineage-relevant information. With the aid of our models, we were able to identify minimal sets of coupling patterns that could replicate, at a given regularization level, observed two-site frequencies in the donor sequences. Analysis of these interactions across species allowed us to identify specific two-site coupling patterns that differentiate plants, animals and fungi. This sequence composition signature was embedded in two-site interactions involving the last nucleotides of the intronic part of the sequences, suggesting that they could be related to taxon-specific features of the early and pre-catalytic spliceosome.

## Supporting information

S1 Text Supplementary Material and Methdos. (PDF)

S2 Text Supplementary Figures. (PDF)
