# Supplementary material for: Conserved and divergent signals in 5’ splice site sequences across fungi, metazoa and plants
Source: PLoS Comput Biol. 2023 Oct 13;19(10):e1011540. doi: 10.1371/journal.pcbi.1011540 (PMC10599564; doi:10.1371/journal.pcbi.1011540)
Supplement: S1 Text — (PDF) [file pcbi.1011540.s001.pdf]

Supplementary Text 1
Conserved and divergent signals in 5' splice site sequences across fungi, metazoa and plants

Maximiliano Beckel[1,2,†], Bruno Kaufman[1,2,†], Yanovsky, Marcelo[1,2*], and Chernomoretz, Ariel[1,3*]

[1]Fundacion Instituto Leloir, Buenos Aires, Argentina
[2]Instituto de Investigaciones Bioquímicas de Buenos Aires, Consejo Nacional de Investigaciones Científicas y Técnicas (CONICET), Buenos Aires, Argentina
[3]Departamento de Fisica, Facultad de Ciencias Exactas y Naturales, Universidad de Buenos Aires, Instituto de Fisica de Buenos Aires, Consejo Nacional de Investigaciones Científicas y Técnicas (CONICET), Buenos Aires, Argentina
[†]Equally contributed

A Maximum Entropy Models

Maximum entropy models are based on information theory principles [1], and are applied to the problem of inferring joint probability functions $P(\mathbf{x})$ satisfying constraints f_ν^{obs} related to $\nu = 1, ..., M$ experimental observations. An estimator $\hat{P}$ for the sought probability distribution is obtained by maximizing the entropy functional $\mathcal{S}$, which is defined in Equation 1:

$$\mathcal{S}[P(\mathbf{x}), \lambda_\nu] = -\sum_{\mathbf{x}} P(\mathbf{x}) \ln\left(P(\mathbf{x})\right) + \lambda_0 \left(\sum_{\mathbf{x}} P(\mathbf{x}) - 1\right) + \sum_{\nu=1}^{M} \lambda_\nu \left(f_\nu^{obs} - \langle f_\nu \rangle_P\right) \quad (1)$$

The first term expresses the Shannon entropy for the distribution, while the second term enforces a normalization constraint on $P(\mathbf{x})$. The third term carries the influence of the deviations between observed frequencies f_ν^{obs} (be they single-site, two-site or otherwise) compared to $\langle f_\nu \rangle_P$, their modelled counterparts as estimated from the modelled probability function $P(\mathbf{x})$. Lagrange multipliers λ_ν weigh different contributions for the constraint corresponding to different observed frequencies (λ_0 merely weighs normalization).

The purview of this study was to estimate $P(\vec{S})$ in regard to an ensemble of $5'$ splicing sites sequences $\{\vec{S}\}$. This ensemble consists of separate $L = 9$-base long sequences as explained in the main manuscript, referred to as $\vec{S} =$

$(s_{-3}, s_{-2}, s_{-1}, s_1, s_2, s_3, s_4, s_5, s_6)$ where $s_i \in \{A, C, G, T\}$. The observed nucleotidic apparition frequencies at each site $f_i(s_i)$, and the two-site correlations between bases $f_{ij}(s_i, s_j)$ are used as model constraints to infer $\hat{P}(\vec{S})$. These observations represent 36 1-site and 576 two-site frequencies, for a total of $M = 612$ fitting parameters in the probabilistic model. The observed frequencies in 1 can thus be expressed as

$$\begin{aligned}\{f_\nu^{obs}\} =& \{f_{-3}(A), f_{-3}(C), ..., f_i(s_i), ...f_{+6}(T),\\ & f_{-3,-2}(A, A), ..., f_{ij}(s_i, s_j), ..., f_{+5,+6}(T, T)\}\end{aligned} \tag{2}$$

so that the entropy functional can be rewritten as shown in Equation 3, where $h_i(s_i)$ and $J_{ij}(s_i, s_j)$ denote the one-site and two-site fitting parameters, respectively:

$$\begin{aligned}\mathcal{S}[P(\vec{S}), \lambda_\nu] = &- \sum_{\vec{S}} P(\vec{S}) lnP(\vec{S}) + \lambda_0 \left(\sum_{\vec{S}} P(\vec{S}) - 1 \right) + \\ & \sum_{i=1}^{L} \sum_{s_i=\{A,C,G,T\}} h_i(s_i) \left(f_i(s_i) - P_i(s_i) \right) + \\ & \sum_{i=1}^{L-1} \sum_{j>i} \sum_{s_i=\{A,C,G,T\}} \sum_{s_j=\{A,C,G,T\}} J_{ij}(s_i, s_j) \left(f_{ij}(s_i, s_j) - P_{ij}(s_i, s_j) \right)\end{aligned} \tag{3}$$

It can be appreciated that the entropy functional in Equation 3 includes terms that penalize deviations between the observed frequencies and the probabilities obtained from the model $P(\vec{S})$. The Lagrange multipliers, $h_i(s_i)$ and $J_{ij}(s_i, s_j)$, weigh the contribution of these deviations in the overall entropy. The goal is to find the values of these fitting parameters that maximize the entropy functional, resulting in the estimated probability distribution $\hat{P}(\vec{S})$. Optimizing this functional leads to the following expression:

$$\frac{\partial \mathcal{S}}{\partial P(\vec{S})} = 0 = -ln(\hat{P}) - 1 + \lambda_0 + \sum_{i=1}^{L} h_i(s_i) + \sum_{i=1}^{L-1} J_{ij}(s_i, s_j) \tag{4}$$

After taking into account the normalization condition: $\sum_{\vec{S}} \hat{P}(\vec{S}) = 1$, Equation 4 implies that:

$$\hat{P}(\vec{S}) = \frac{1}{Z} e^{-E_d(\vec{S})} \tag{5}$$

with

$$E_d(\vec{S}) = -\sum_{i=1}^{9} h_i(s_i) - \sum_{i<j}^{9} J_{ij}(s_i, s_j) \tag{6}$$

where $Z = \sum_{\vec{S}} e^{-E_d(\vec{S})}$ is a normalization constant.

After estimating the fitting parameters (see Section B of the supplementary material) the estimated $\hat{P}$ should fulfill:

$$\hat{P}_i(s_i) \equiv \sum_{\forall j \neq i} \sum_{s_j} \hat{P}(\vec{S}) \quad \sim \quad f_i(s_i) \tag{7}$$

$$\hat{P}_{i,j}(s_i, s_j) \equiv \sum_{\forall k,l \neq i,j} \sum_{s_k, s_l} \hat{P}(\vec{S}) \quad \sim \quad f_{ij}(s_i, s_j) \tag{8}$$

B Fitting procedure

The technique of gradient descent was used to fit 612 the parameters which constitute our model. Each iteration generated an ensemble of 100000 sequences via a Metropolis-Hastings rejection sampling scheme compatible with the probability distribution function described by Eq. 5, using the current model parameters. The iteration then updated said parameters according to the following rules:

$$h_i^{t+1} \leftarrow h_i^t - \epsilon_h [f_i - \hat{P}_i] \tag{9}$$

If $J_{ij}^t = 0$:

$$J_{ij}^{t+1} \leftarrow \begin{cases} 0 & \text{if } |\Delta| < \gamma \\ \epsilon_j [\Delta - sign(\Delta)] & \text{if } |\Delta| > \gamma \end{cases} \tag{10}$$

If $J_{ij}^t \neq 0$:

$$J_{ij}^{t+1} \leftarrow \begin{cases} J_{ij}^t + \epsilon_j [\Delta - \gamma sign(J_{ij}^t)] & \text{if } \eta > 0 \\ 0 & \text{if } \eta < 0 \end{cases} \tag{11}$$

with $\Delta = f_{ij} - \hat{P}_{ij}$ and $\eta = (J_{ij}^t + \epsilon_j [\Delta - \gamma sign(J_{ij}^t)]) * J_{ij}^t$.

The regularization parameter γ embodies the role of a threshold preventing weaker influences from creating nonzero coupling parameters, and extinguishing coupling parameters when their associated two-site probabilites are weak enough.

C The statistical model

A family of models was fitted for each analyzed species. As the regularization parameter γ was decreased, models increased their complexity in a controlled fashion. This strategy thus yielded minimal sets of coupling constants J_{ij} necessary to adjust the observed two-site frequencies f_{ij} at a given accuracy level. This behavior is demonstrated in Fig A (specifically for the case of *Homo sapiens*.

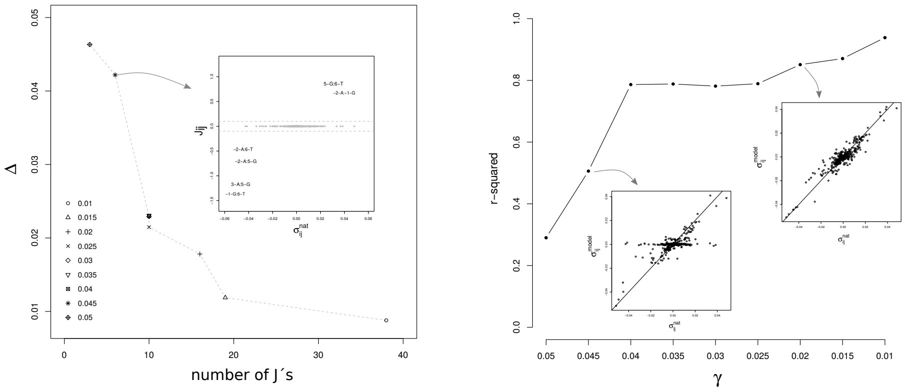

Fig A **Role of regularization**. Left panel: maximum absolute deviation between observed and estimated two site probabilities, $\Delta = max[abs(f_{ij} - P_{ij})]$, as a function of the number of non-zero coupling constants identified at different regularization levels (different point shape, $\gamma \in [0.01, 0.05]$) for human donor sequence models. Inset: coupling constants $J_{ij}(s_i, s_j)$ as a function of observed two-site correlations $\sigma_{i,j}^{nat}$ for the $\gamma = 0.045$ model. Right panel: r^2 correlation coefficient between modeled and observed two-site correlations as a function of the regularization parameter γ. Insets: relationship between modeled, $\sigma_{i,j}^{model}$ and observed, $\sigma_{i,j}^{nat}$, two-site correlations at $\gamma = 0.045$ and $\gamma = 0.02$.

The left panel of Fig A shows $\Delta = max[abs(f_{ij} - P_{ij})]$, an indicator of the deviation between two-site observed frequencies and their respective estimated probabilities, as a function of the number of non-zero coupling parameters obtained by the model at a given γ regularization level. We can observe that a weaker regularization results in a more complex model that better fits target observed frequencies. The inset shows coupling parameter values J_{ij} as a function of the corresponding correlations observed in natural sequences for a specific regularization value ($\gamma = 0.045$). Only six non-trivial parameters are contained by this specific model, and they correspond to the largest two-site correlations present in the data.

At every regularization level the fitted models were able to generate an ensemble of sequences presenting one-site P_i and two-site P_{ij} which accurately reproduced observed one-site and two-site frequencies (R-squared > 0.99). In the right panel of Fig A we can visualize the coefficient of determination (R-squared) for the regression of model-estimated against observed correlation values, as a function of the regularization level employed. As the algorithm allows more couplings to emerge (weaker regularization), models more accurately reproduce two-site correlations present in natural sequences. A similar behavior surfaces for all analyzed genomes (see B). From said figure we may notice that models

obtained at a regularization level of $\gamma = 0.025$ presented around ten coupling parameters different from zero, and achieved a noticeable reduction in Δ. On the other hand, a leveling-off in Δ was consistently observed at $\gamma = 0.015$. This implies diminishing returns within this range.

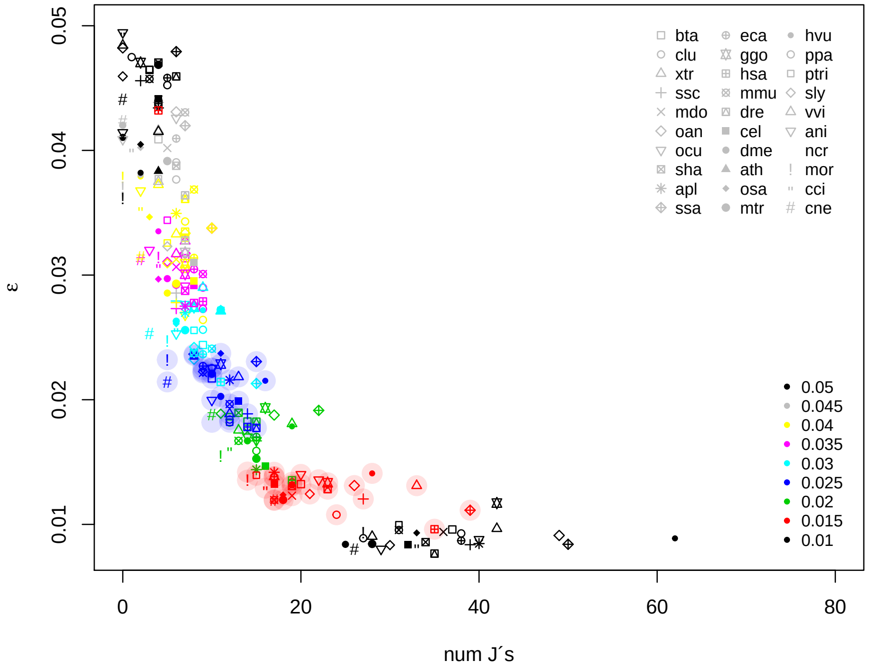

Fig B **Model convergence.** Maximum absolute deviation between observed and estimated two site probabilities, $\Delta = max[abs(f_{ij} - P_{ij})]$, as a function of the number of the non-zero coupling parameters identified at different regularization levels (different colors, $\gamma \in [0.01, 0.05]$). Results for different analyzed organisms are depicted with different point shapes (see legend). Shaded symbols highlight $\gamma = 0.025$ (blue) and $\gamma = 0.015$ (red) models respectively.

To better understand the roles and patterns of the fitting parameters $\{h_i\}$ and $\{J_{ij}\}$, Fig C shows a graphical summary of the $\gamma = 0.025$ model for human donor sequences. The left panel shows a relationship between estimated and observed one-site and two-site frequencies. Correlation is easily observed between natural frequencies and those produced by the fitted model (R-squared > 0.99 for both one-site and two-site probabilities). An inset in this panel shows the relationship between modeled and observed correlations. At a given level of regularization, said correlations were somewhat harder to reproduce (R-squared~ 0.84). The fitting procedure only considers up to level-two marginal probabilities (i.e. f_i and f_{ij}), and yet we found that the inclusion of a finite set of pairwise couplings J_{ij} allowed this model to reproduce higher-order statistics.

Three-site probabilities (f_{ijk}, gray dots in FigC) were adequately captured in the simulated ensemble of sequences (R-squared = 0.95).

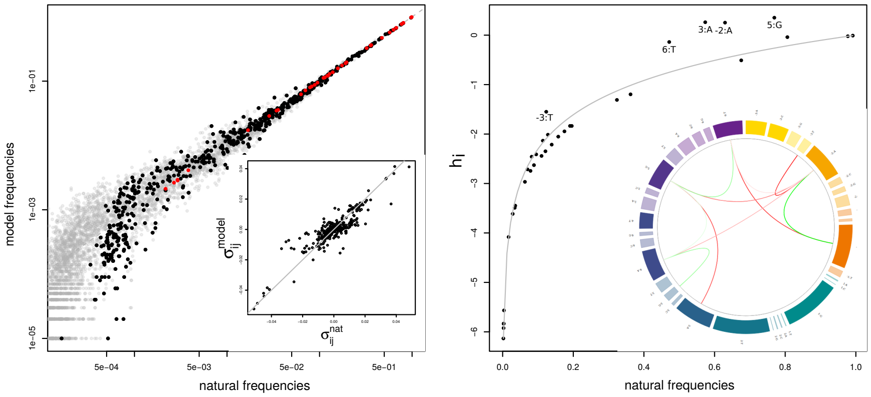

Fig C **Model frequencies and fitted parameters at $\gamma = 0.025$.** Left panel: one-site, two-site and three-site model estimated frequencies (p_i, p_{ij}, p_{ijk}) as a function of their corresponding observed frequencies (f_i, f_{ij}, f_{ijk}), depicted as red, black and gray dots respectively. Inset: relationship between model-estimated, σ_{ij}^{model}, and observed, σ_{ij}^{nat} two-site correlations. Right panel: $h_i(s_i)$ fitting values as a function of their observed one-site frequencies, $f_i(s_i)$. The continuous gray line depicts the logarithmic relationship expected by the independent site model, $h_i^{indep}(s_i) \sim \log(f_i(s_i))$. Inset: circos representation of fitted model. A ring of 36 boxes shows nine sites in different colors, and four boxes within each to represent the four possible nucleotide occurrences A, C, G, T. Warm colors were used for the three exonic sites whereas cold colors for intronic ones. The area of each box is proportional to the observed nucleotide-site probability $f_i(s_i)$. Positive and negative couplings between site-base occurrences are depicted with green and red lines respectively.

The right panel of Fig C shows h_i parameter values as a function of observed frequencies f_i; said relationship approximately obeys a logarithmic function, shown in grey. Deviations from this curve are due to the indirect influence of two-site interactions, as the simple logarithmic relationship corresponds to a model with independent sites. As two-site coupling parameters emerge, single-site parameters must adjust in order to preserve single-site statistics while also accomodating two-site observed frequencies. Positive deviations occur whenever the site is involved in negative interactions, and vice-versa. For instance, the single-site parameters $h_5(G)$ and $h_6(T)$ present larger values than would be expected from a single-site model; this is caused by a positive interaction between them, stabilizing consensus G and T bases at the two last intron sites.

The inset in this panel shows a *circos* graphical representation of the model fitted in this example. The outer ring summarizes single-site statistics: each

separate color represents a given site on sequences (cool colors represent six intronic sites, whereas warm colors correspond to the exon). Each site is divided into four boxes: their relative sizes represent the occurrence of $\{A, C, G, T\}$ bases in single-site sequence statistics. Positive and negative couplings between different site-base occurrences are depicted with green and red lines respectively.

D Model validation

In order to validate our model, a good approach would be to use it to recover a-priori known statistical interactions between site-base occurrences. Unfortunately, no literature studies provide a reliable set of ground-truth interactions available for validation tasks. The following subsection will show the alternative: a simulation-based framework constructed in order to test the ability of our maximum-entropy models to uncover known significant statistical patterns embedded in sequence composition. Afterwards, the next subsection presents a cross-validation analysis as well as an assessment of robustness against noisy data.

D.1 Analysis of simulated ensemble of sequences

An important part of model validation was to corroborate the recovery of known parameters from data. In order to evaluate this, we generated an artificial ensemble of sequences using the parameters obtained by the $\gamma = 0.025$ model for metazoan organisms (middle column of Fig 4 in the main manuscript). Said ensemble was produced through a generative process based on Bayesian Networks (BN) in such a way as to maintain compatibility with one-site (36 parameters) and two-site (7 pairwise interactions) statistical patterns. BNs are graphical representations of joint probability distributions that use directed graphs to explicitly encode a set of statistical dependence assertions among random variables. This case concerned 9 random variables, each associated with a position of the donor sequence.

The first row of Fig D shows the undirected graph representing the statistical interactions to be embedded in simulated sequences, along with corresponding one-site marginal probabilities. We can notice a large connected component of seven nodes, and two more isolated nodes.

The BN shown in the middle row was used to generate nucleotidic sequences according to the following Monte Carlo process: First, a base for site -3 was chosen at random following the corresponding one-site probabilities, $P(-3)$. Then, the base for site -2 was chosen according to a conditional one-site probability, $P(-2|-3)$, that depended on the realization of the random choice that took place at the precedent step for site -3. In this case, if a T nucleotide was obtained at position -3, the probability of generating an A at position -2 was penalized by a factor ϵ to account for the negative interaction between these two sites. This Monte Carlo procedure continued randomly choosing nucleotides for sites -1 and 5 (with probabilities conditioned on the outcome of

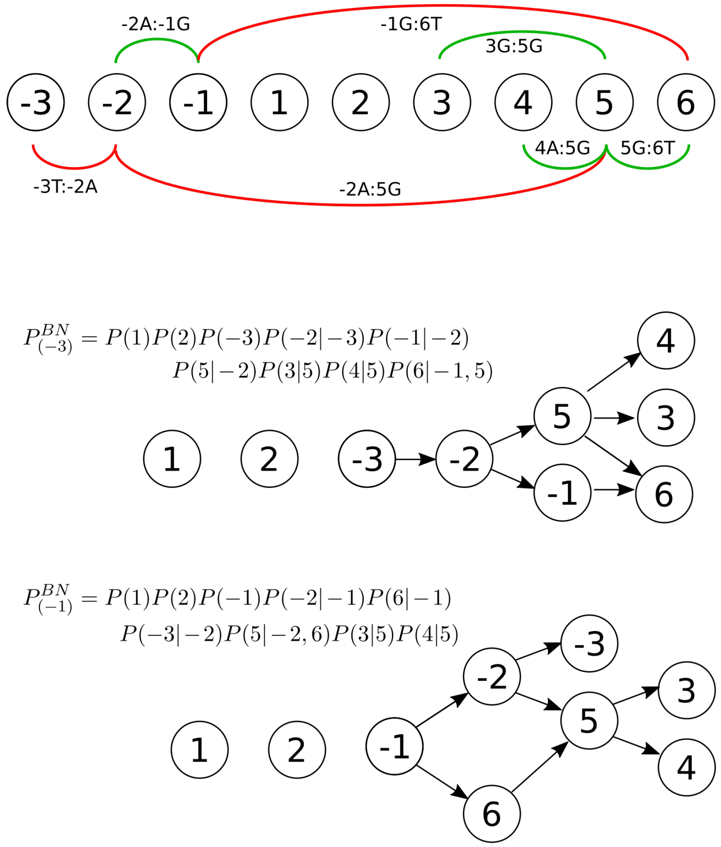

Fig D **Bayesian network-based generative procedure.** The embedded interaction pattern used to validate our model is shown in the first row. Green and red arcs correspond to positive and negative interactions respectively. In the middle and bottom rows we show Bayesian networks used to generate sequences starting from sites -3 and -1 respectively.

site -2), sites 4 and 3 (conditioned by the random occurrence that took place at site 5) and then site 6 (which depended on the results obtained at sites -1 and 5). Finally, bases were assigned to the isolated (i.e. statistically independent) nodes at sites 1 and 2, according to their corresponding one-site probabilities. Sequences generated with this procedure were compatible with the joint probability distribution shown by the middle panel figure.

The bottom panel of Fig D displays another BN that could have also been considered to generate random sequences. In this case the Monte Carlo procedure begins at site -1 and then sequentially follows the directed acyclic graph shown in the figure. Overall, there existed seven different BNs that could be used to generate random sequences, all of them compatible with the interaction pattern represented in the first row of the figure. Each one of them starts from one of the seven different nodes belonging to the largest component of the interaction graph.

With these tools at hand, we simulated an ensemble of 500000 sequences by repeatedly choosing one of these BNs at random and following the associated procedure to sample the corresponding nucleotides. A $\gamma - 0.025$ maximum entropy model identified 3 positive and 2 negative coupling parameters that matched the pairwise correlation structure embedded in the simulated sequences. Reducing the regularization level (i.e. considering a $\gamma - 0.01$ model) resulted in the identification of 4 positive and 4 negative coupling constants.

To better understand the performance of our model strategy, Fig E shows the ranking of the top 200 pairwise correlations present in the simulated dataset. Positive coupling constants identified by our models are shown in green, while negative ones are displayed in red. Black circles surround those identified by a $\gamma = 0.025$ model, while uncircled colored points indicate those identified by the $\gamma = 0.01$ model. Small, uncolored circles show correlations with no interaction parameter obtained by the model. The goal of our validation methodology was to recover the seven interactions labeled in black letters. The more strongly regularized model at $\gamma = 0.025$ recovered five parameters within this group, and only those parameters. The more weakly regularized model at $\gamma = 0.01$ recovered all seven target interactions, but also recovered a greyed-out interaction between the $3A$ and $5G$ occurrences. This shows that as regularization is relaxed, our models can accurately and progressively recover statistical information embedded in sequence ensembles.

Comparing Fig E with the top panel of Fig D we may verify that our simulation strategy successfully introduced the desired correlation patterns in the sequence ensemble, while the model validation strategy successfully recovered the pairwise correlation structure embedded in the data.

D.2 Cross validation

Cross-validation was performed using the human donor sequence ensemble as a basis. Said sequences were placed into ten equally-sized bins according to their appearance frequency within the genome. We then randomly sampled 10% of sequences from each bin in order to construct a representative validation set. Using the remaining 90% of sequences as a training set, a model was fitted using a regularization level of $\gamma = 0.025$. Fig F displays the relationship between the frequency of validation sequences and their estimated energy values, $E_{90\%}$, calculated using parameters fitted by the training dataset exclusively. An inset shows the relationship between $E_{90\%}$ and $E_{100\%}$, the sequence energies when using parameters fitted to a complete dataset.

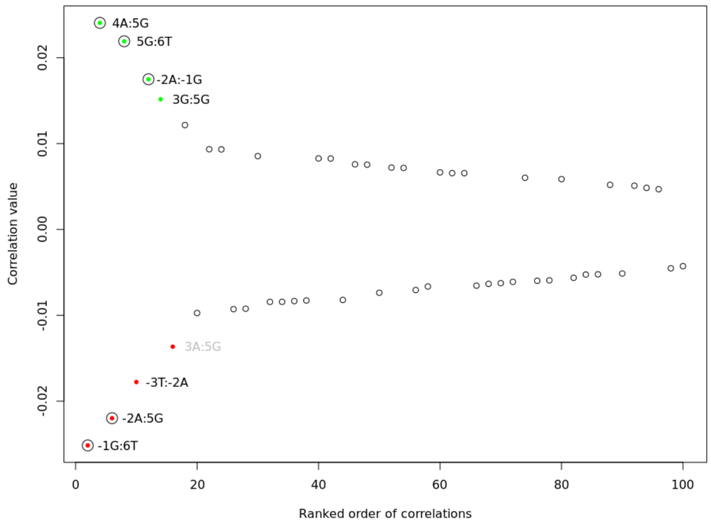

Fig E **Gold-standard interaction recovery.** Top 200 ranked pairwise correlation values for simulated sequences, ranked in order of absolute magnitude. Black text labels are used to point out interactions explicitly embedded within our simulated ensemble, while a greyed out interaction between $3A$ and $5G$ occurrences was not explicitly placed within the data, but nonetheless obtained by a weakly regularized model. Five correct parameters recovered by a $\gamma = 0.025$ model are shown in circles, while those recovered by a $\gamma = 0.010$ are shown uncircled. Green circles show recovered interaction parameters of a positive nature, while red shows those which are negative. Uncolored circles denote correlations whose interaction parameters were proposed by neither model.

We can observe that estimated energies, $E_{90\%}$, for unseen validation sequences closely follows the decreasing relationship expected in regard to appearance frequency. As the inset shows, energy calculation is largely unaffected when the model is trained with incomplete data.

D.3 Robustness against noise

In order to generate a progressively noisy dataset, our basis was the $\gamma = 0.025$ generative model obtained for *H. sapiens*, containing 11 non-null two-site interaction parameters $J_{ij}(s_i, s_j)$. This was used to generate an ensemble of 100000 9nt sequences. Five different additional ensembles were constructed by producing, on average, a random point mutation each 100, 20, 10, 5, and 2 sequences respectively. Each noisy ensemble was used to fit a separate model, and said models were assessed by comparing the newly obtained parameters with those originally found for H. sapiens.

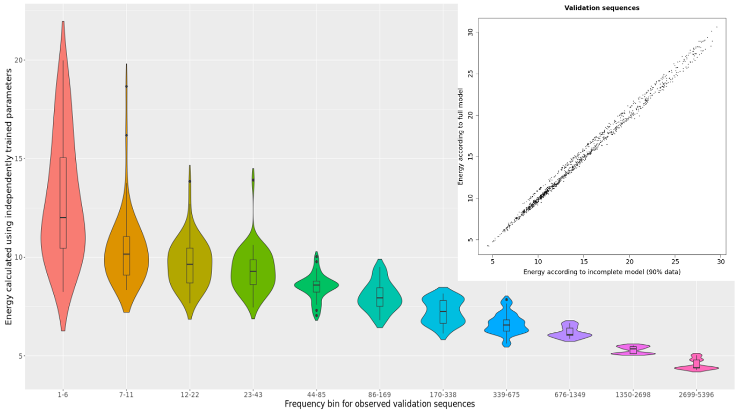

Fig F **Sequence energy consistency despite incomplete training.** Energy ($E_{90\%}$) violin plots for validation sequences sorted into equally populated frequency bins. The inset displays the relation between $E_{100\%}$ and $E_{90\%}$ energy values estimated for validation sequences when using parameters from models trained on full data and on the 90% training dataset respectively.

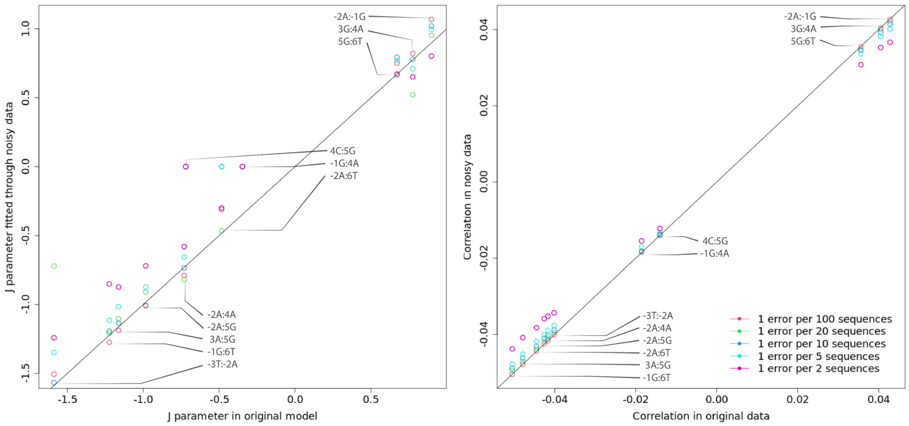

Fig G **Parameter fitting through noisy data.** Coupling parameters (left panel) and correlation values (right panel) estimated for different noisy ensembles as a function of the corresponding values obtained using the original H. sapiens dataset.

As shown in the left panel of Fig G, the maximum entropy model was able to recover 9 out of 11 interactions for almost all cases, despite the presence of noise which only scaled down the effective value of the coupling parameter. The coupling constants 4C:5G and -1G:4A could not be recovered at all. To further understand these results, we analyzed the pairwise correlations in the noisy and original datasets, as illustrated in the right panel of Fig G. Correlations were

incrementally degraded by increasing noise levels. It is evident that the pairwise correlations for -1G:4A and 4C:5G had the lowest values in the original dataset and were particularly sensitive to said noise. These signals severely weakened in the noisy sequence ensembles: this caused the corresponding parameters to not appear in their regularized fitted models.

In conclusion, based on our analyses of gold-standard retrieval, cross-validation, and robustness, we believe that our modeling approach effectively, accurately and robustly uncovers pairwise statistical couplings embedded in the training datasets.

E Analysis of 3'ss

Each annotated human donor splicing site has its counterpart: an acceptor site on the other side of its corresponding intron. In order to analyze possible statistical interactions between the donor and acceptor site, we concatenated each 9-nt donor sequence with a 13-nt long sequence from its corresponding acceptor site (12 intronic and 1 exonic sites). We thus constructed 22-nt long sequences to analyze through our maximum entropy model fitting strategy, training a $\gamma = 0.025$ model with this ensemble. Through its obtained parameters were able to characterize interactions within acceptor and donor sites, and a lack of interactions between them.

The circos picture included in Fig H summarizes the detected two-site coupling patterns for this extended dataset. Our analysis did not find any statistical evidence supporting a connection between 5'ss donor sites and 3'ss acceptor sites. The detected coupling pattern within donor sites was almost identical to those originally reported in the manuscript, showing further consistency in our approach. These findings validate the strategy used to uncover biologically relevant statistical patterns from 5'ss.

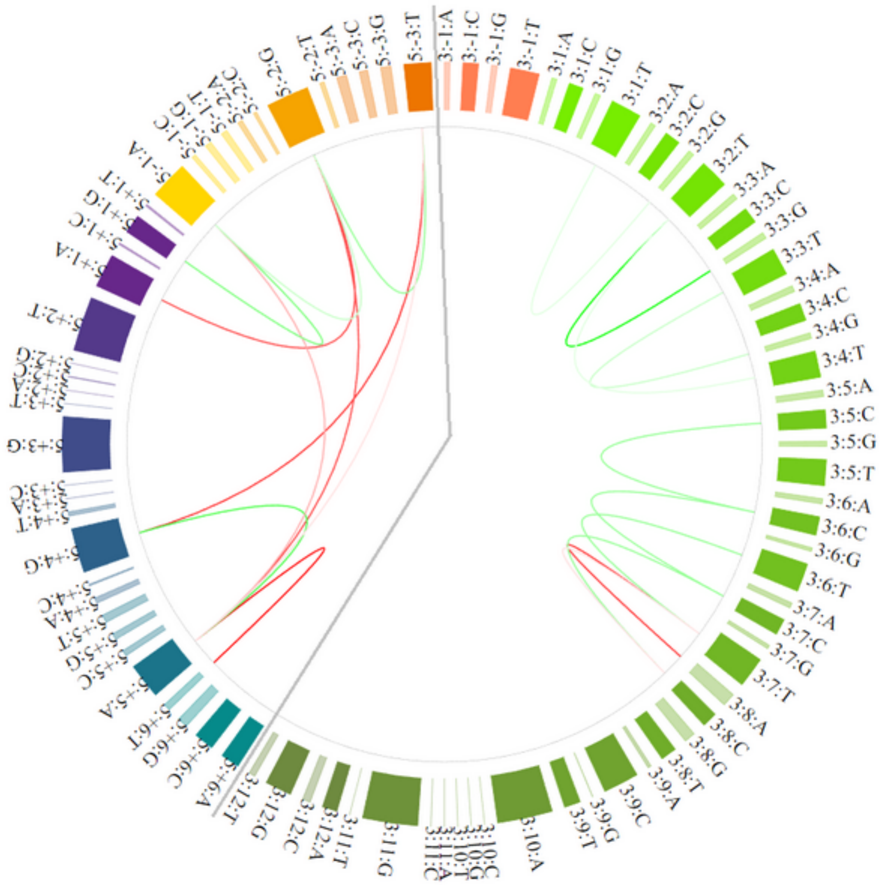

Fig H **Joint statistical pattern including donor and acceptor sites.** Two-site coupling patterns detected for the ensemble of extended splicing sequences composed by 9 (3 exonic and 6 intronic) donor sites, followed by 13 (12 intronic and 1 exonic) acceptor sites. Each box represents the observed frequency of a given nucleotide at a given site. Exonic and intronic sites were colored using warm and cold color palettes respectively. Red and green curves represent negative and positive interactions between site-base combinations respectively. The gray line visually separates donor and acceptor sites, and shows that no interactions cross between the 5'ss and 3'ss regions.

F Energy landscape

To further characterize the energy estimated for donor sequences, we analyzed the $\gamma = 0.025$ model for human donor sequences through a network graph approach. Representing individual sequences with nodes, two were connected if and only if their sequences only differed by one mutation (i.e. a Hamming

distance of 1). These edges were directed in a single way, going from the higher-energy node to its lower-energy counterpart. This procedure constructed an energy landscape in the shape of a network, where one sequence could lead towards another, but only by existing at a distance of one mutation and by undergoing an energetically convenient transition.

In this directed network, we found 243 sequences corresponding to local energy minima; this is to say, these nodes displayed no outwards edges (i.e. null out-degree), and thus were terminal states for all transitions. For each one of these attractor-nodes we estimated the inward connected component, i.e. the set of nodes that could reach this given local minimum-energy node in a finite number of steps. These sets of nodes describe the extent of the basin of attraction of a given attractor sequence.

The first violin in Fig I corresponds to a huge main basis of attraction, which contained 9593 sequences. This number represented 80% of the complete set of 5' exon-intron boundaries of the *H. sapiens* genome, and 96% of the entire set of GT 5'ss. The attractor's minimum-energy sequence $\vec{S}^* = \{C, A, G, G, T, A, A, G, T\}$ is the perfect matching sequence of U1 smRNA and presents an energy value $E_d(\vec{S}^*) = 3.5$. We can see from the figure that the basins of attraction of the rest of the local minima presented much higher energy values in far shallower energy wells. Among these secondary local minima, there were only 14 attractors presenting GT as first intron nucleotides.

These results suggest that, in terms of the modelled data-driven energy, our approach presents a well-defined global minimum state laying inside a wide energy well.

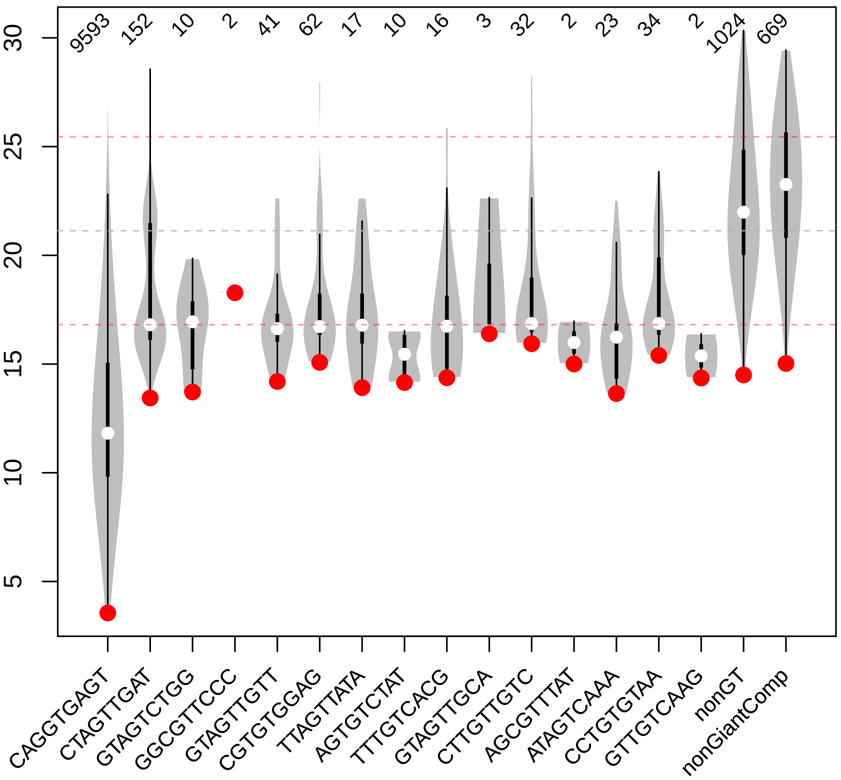

Fig I **Energy landscape characterized through basins.** Violin plots for the energy distribution of sequences belonging to different basins of attraction. Each violin plot shows the lowest and highest energies within the corresponding basin and summarizes the overall energy distribution. The first 15 violins correspond to basins whose minimum-energy sequence presents a GT-dinucleotide where common, at the start of the intron. The two last violins correspond to a cumulative set of non-GT attractor sequence basins, and another set of sequences that did not belong to the giant component of the graph at all. Red dots highlight the attractor (i.e. minimal) energy of each basin. The corresponding sequence is included as an x-axis label. The top label shows the number of sequences contained in each basin.

G Fowlkes-Mallows analysis

Given two different partitions of n objects, and being $m_{i,j}$ the number of common elements between the i-th and j-th clusters of the first and second partitions respectively, the Fowlkes-Mallows index is defined as:

$$B_k = \frac{T_k}{\sqrt{P_k Q_k}}$$

where

$$T_k = \sum_{i=1}^{k}\sum_{j=1}^{k} m_{i,j}^2 - n \quad (12)$$

$$P_k = \sum_{i=1}^{k}(\sum_{j=1}^{k} m_{i,j})^2 - n \quad (13)$$

$$Q_k = \sum_{j=1}^{k}(\sum_{i=1}^{k} m_{i,j})^2 - n \quad (14)$$

and $0 \leq B_k \leq 1$. A higher value for the Fowlkes–Mallows index indicates a greater similarity between the clusters of the two compared partitions.

K-cut partitions were produced for phylogenetics and P_{ij}-induced dendrograms. For each k value the Fowlkes-Mallows clustering similarity index, B_k, was estimated along with 10000 null-model values obtained by label shuffling.

As can be seen from the figure, Fowlkes-Mallows indices were found to be statistically significant ($p_v < 10^{-4}$) for almost the entire range of k-cluster groups ($2 < k < 29$).

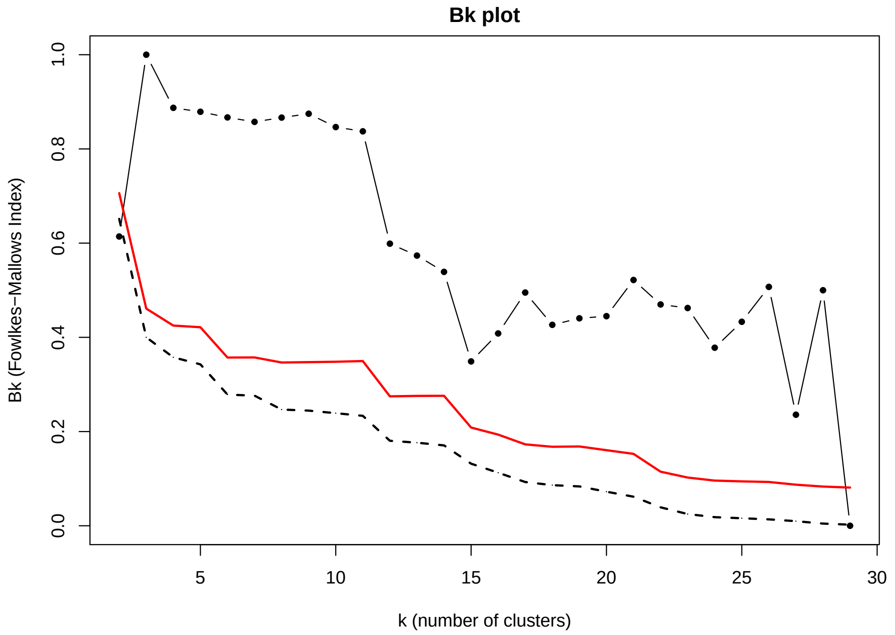

Fig J **Fowlkes-Mallows analysis.** The dotted black line corresponds to observed B_k values as a function of the number of clusters k. The expected values under the null hypothesis of no relation between clusterings are shown as a dashed black line. The full red line depicts the 95% one-side rejection region based on the asymptotic distribution of B_k values. All calculations were performed using the *Bk_plot* function implemented within the R package *dendextend* [2].

References

[1] Jaynes E. T. (1957) Information theory and statistical mechanics. Physical Review, 106, 620-630. doi:10.1103/PhysRev.106.620

[2] Galil, T.(2015). dendextend: an R package for visualizing, adjusting, and comparing trees of hierarchical clustering. Bioinformatics, doi:10.1093/bioinformatics/btv428
