# Supplementary material for: Conserved and divergent signals in 5’ splice site sequences across fungi, metazoa and plants
Source: PLoS Comput Biol. 2023 Oct 13;19(10):e1011540. doi: 10.1371/journal.pcbi.1011540 (PMC10599564; doi:10.1371/journal.pcbi.1011540)
Supplement: S2 Text — (PDF) [file pcbi.1011540.s002.pdf]

Supplementary Text 2

Conserved and divergent signals in 5' splice site sequences across fungi, metazoa and plants

organism	code	assembly	5'ss (unique)	5'ss GT (unique)
aspergillus nidulans	ani	ASM1142v1	24563 (3954)	24514 (3919)
coprinopsis cinerea okayama	cci	CC3	61427 (4737)	61325 (4670)
cryptococcus neoformans	cne	cryp_neof_125_91_V1	36046 (2637)	35220 (2435)
magnaporthe oryzae	mor	MG8	22772 (3126)	22567 (3039)
neurospora crassa	ncr	NC12	16739 (2522)	16533 (2423)
arabidospis thaliana	ath	TAIR10	136036 (11286)	130069 (6803)
hordeum vulgare	hvu	IBSCv2	499846 (48879)	413160 (10107)
medicago truncatula	mtr	MedtrA17_4.0	156335 (11396)	148511 (6964)
oryza sativa	osa	IRGSP-1.0	130702 (12688)	124646 (8418)
physcomitrium patens	ppa	Phypa V3	194684 (8310)	191147 (6991)
populus trichocarpa	ptri	Pop_tri_v3	179803 (10168)	176766 (9285)
solanum lycopersicum	sly	SL3.0	141263 (12562)	135225 (8400)
vitis vinifera	vvi	12X	108097 (9614)	105266 (7713)
anas platythynchos platyrhynchos	apl	CAU_duck1.0	152272 (9075)	146465 (5405)
bos taurus	bta	ARS_UCD1.2	203086 (11919)	194432 (6624)
canis lupus familiaris	clu	CanFam3.1	215912 (12437)	206340 (5521)
danio rerio	dre	GRCz11	276776 (11723)	268477 (6720)
equus caballus	eca	EquCab3.0	216007 (10367)	208399 (4984)
gorilla gorilla	ggo	gorGor4	212738 (14620)	200262 (7697)
homo sapiens	hsa	GRChv38	502197 (12129)	488939 (6206)
monodelphis domestica	mdo	ASM229v1	208511 (14090)	196940 (6970)
mus musculus	mmu	GRCm39	391997 (9026)	384141 (5369)
ornythorhyncus anatinus	oan	mOrnAna1.p.v1	187119 (13517)	175225 (6562)
oryctolagus cuniculus	ocu	OryCun2.0	147142 (12204)	138124 (5217)
sarcophilus harrisii	sha	mSarHar1.11	227688 (10104)	220325 (5637)
salmo salar	ssa	ICSASGv2	516921 (28445)	455502 (14209)
sus scrofa	ssc	Sscrofa11.1	260642 (21094)	243064 (8300)
xenopus tropicalis	xtr	Xenopus_tropicalis_v9.1	231618 (20862)	205430 (10794)
caenorhabditis elegans	cel	WBcel235	127661 (7625)	124619 (5351)
drosophila melanogaster	dme	BDGP6.32	63121 (4023)	62451 (3780)

Table A **Analyzed genomes.** For each analyzed species we show its associated assembly code, the total number of 5' exon-intron boundaries, the number of different 5'ss sequences and the number of different 5'ss sequences presenting GT as the first two intronic bases.

Species	IC-EC	IC-ENC	INC-EC	INC-ENC	IC-IC	IC-INC	INC-INC	EC-EC	EC-ENC	ENC-ENC
**cne**	-0.41	0	0	0	-0.14	-0.01	0	0.88	0.13	-0.13
**ani**	-0.5	0	0.03	0	0.21	0.02	0	0.82	0	-0.15
**ncr**	-0.68	0	0	0	0.26	0.03	0	0.68	0	-0.07
**mor**	-0.45	0	0	0	0.1	0	0	0.87	0	-0.15
**cci**	-0.59	0	0.04	0	0.15	0.09	0	0.7	0	-0.35
**ath**	-0.39	0	0.05	0	0.37	0	0	0.83	0	-0.11
**hvu**	0	0.09	0	-0.06	-0.13	0.92	0.09	0	-0.16	-0.29
**mtr**	-0.3	0	0	0	0.1	0	0	0.95	0	0
**osa**	-0.24	0	0.01	0	0.23	0.05	-0.04	0.94	0	0
**ppa**	-0.77	0	-0.01	0	-0.02	-0.01	-0.03	0.64	0	-0.08
**ptri**	-0.42	0	0	0	-0.02	0	0	0.91	0	0
**sly**	-0.36	0	-0.01	0	0.21	0	0	0.91	0	-0.07
**vvi**	-0.37	0	0	0	-0.02	0	0	0.93	0	-0.04
**apl**	-0.4	0	0	0	0.29	0.01	0	0.87	0	0
**bta**	-0.28	0	0	0	0.57	0	0	0.78	0	0
**clu**	-0.3	0	0	0	0.52	0.03	0	0.79	0	0
**dre**	-0.45	0	0	0	0.23	0	-0.02	0.86	0	0
**eca**	-0.37	0	0	0	0.48	0	0	0.8	0	0
**ggo**	-0.18	0	0	0	0.59	-0.01	0	0.78	-0.1	0
**hsa**	-0.48	0	0	0	0.14	-0.01	0	0.86	0	0
**mdo**	-0.22	0	0	0	0.67	0	0	0.69	-0.15	0
**mmu**	-0.51	0	0	0	0.08	0	0	0.86	-0.06	0
**oan**	-0.19	0	0	0	0.81	-0.06	0.03	0.55	0	0
**ocu**	-0.24	0	0	0	0.61	0	0	0.76	0	0
**sha**	-0.33	0	0	0	0.6	0.01	0	0.73	-0.05	0
**ssa**	0.14	0	-0.06	0	0.74	0.01	0.02	0.63	-0.16	0
**ssc**	-0.14	0	0	0	0.7	-0.04	0	0.7	-0.07	0
**xtr**	0.11	0	-0.04	0	0.56	-0.02	0.03	0.81	-0.13	-0.04
**dme**	-0.89	0	0	0	-0.23	-0.04	0	0.38	0	-0.08
**cel**	-0.9	0	0.05	0	-0.21	-0.01	0	-0.35	0	-0.13

Table B **Conserved patterns ($\gamma = 0.025$).** Mean interactions between different groups of site-base occurrences, for different species. EC, ENC, IC, and INC stand for exonic-consensus, exonic-non-consensus, intronic-consensus and intronic-non-consensus occurrences respectively.

Species	IC-EC	IC-ENC	INC-EC	INC-ENC	IC-IC	IC-INC	INC-INC	EC-EC	ENC-EC	ENC-ENC
**cne**	-0.08	0	0	0	0.34	0	0	0.92	0	-0.17
**ani**	-0.48	0	0	0	0.06	0	0	0.88	0	0
**ncr**	-0.56	0	0	0	0.26	0	0	0.79	0	0
**mor**	-0.29	0	0	0	0	0	0	0.96	0	0
**cci**	-0.28	0	0	0	0.24	0.04	0	0.93	0	0
**ath**	-0.37	0	0.02	0	0.19	0	0	0.91	0	0
**hvu**	0	0.13	0	-0.08	0	0.94	0.19	0	0	-0.23
**mtr**	-0.56	0	0	0	0.07	0	0	0.82	0	0
**osa**	-0.22	0	0	0	0.07	0.02	-0.03	0.97	0	0
**ppa**	-0.84	0	0	0	0.05	0	-0.03	0.55	0	0
**ptri**	-0.42	0	0	0	0.02	0	0	0.91	0	0
**sly**	-0.33	0	0	0	0.23	0	0	0.92	0	0
**vvi**	-0.34	0	0	0	0.01	0	0	0.94	0	0
**apl**	-0.41	0	0	0	0.39	0	0	0.83	0	0
**bta**	-0.27	0	0	0	0.59	0	0	0.76	0	0
**clu**	-0.33	0	0	0	0.56	0	0	0.76	0	0
**dre**	-0.34	0	0	0	0.24	0	0	0.91	0	0
**eca**	-0.36	0	0	0	0.51	0	0	0.78	0	0
**ggo**	-0.22	0	0	0	0.57	0	0	0.79	0	0
**hsa**	-0.55	0	0	0	0.16	0	0	0.82	0	0
**mdo**	-0.19	0	0	0	0.57	0	0	0.8	0	0
**mmu**	-0.58	0	0	0	0.12	0	0	0.81	0	0
**oan**	-0.23	0	0	0	0.61	0	0	0.76	0	0
**ocu**	-0.27	0	0	0	0.59	0	0	0.76	0	0
**sha**	-0.31	0	0	0	0.63	0	0	0.71	0	0
**ssa**	0.11	0	-0.05	0	0.61	0	0	0.78	0	0
**ssc**	-0.18	0	0	0	0.62	0	0	0.77	0	0
**xtr**	0.17	0	-0.05	0	0.59	0	0	0.79	0	0
**dme**	-0.97	0	0	0	-0.26	0	0	0	0	0
**cel**	-0.97	0	0.06	0	-0.21	0	0	0	0	-0.1

Table C **Conserved patterns** ($\gamma = \mathbf{0.015}$)**.** Mean interactions between different groups of site-base occurrences, for different species. EC, ENC, IC and INC stand for exonic-consensus, exonic-non-consensus, intronic-consensus and intronic-non-consensus occurrences respectively.

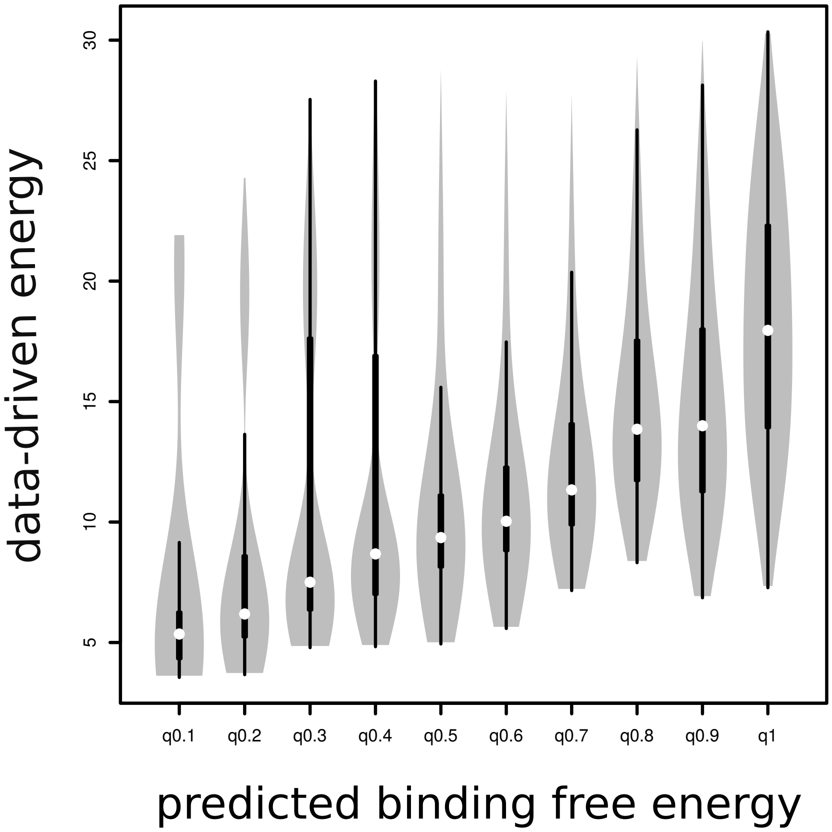

Fig A **Data driven energy.** Boxplots of data-driven energy values estimated for 502197 human 5'ss sequences for every decile of predicted dimerization energies against U1 smRNA. Dimerization free energy between the sequences of the donor sites and the complementary portion of the snRNA U1 was estimated using the program RNAcofold in the ViennaRNA 2.0 package[**?**], using default parameters.

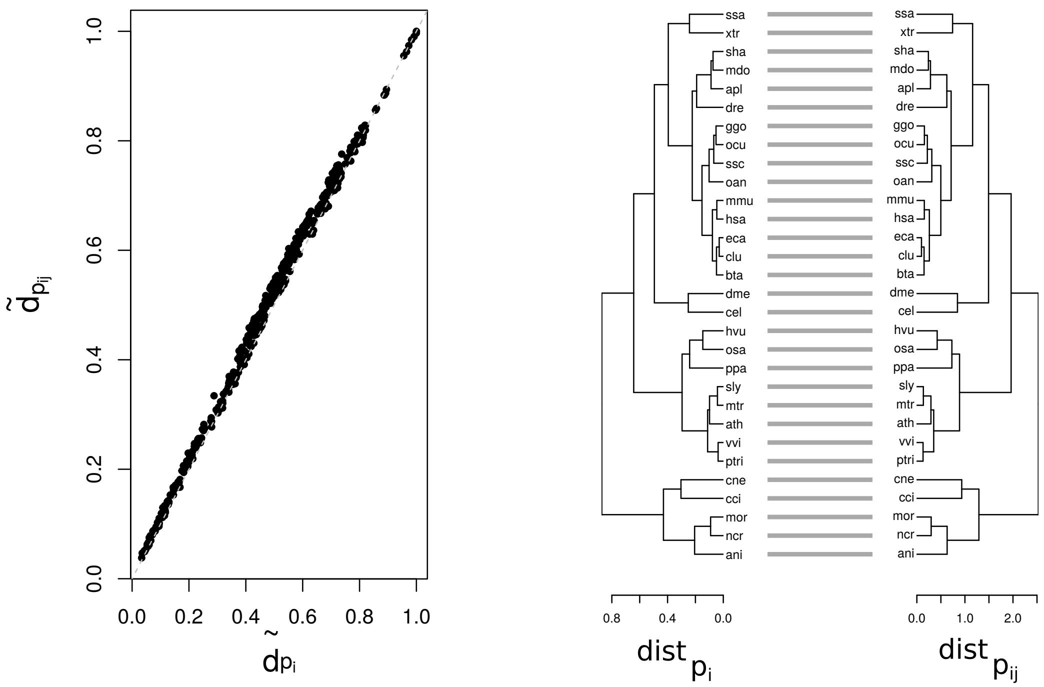

Fig B **Distances inferred from one-site and two-site statistics.** The left panel shows pairwise scaled Euclidean distances between species estimated using P_i (x-axis) and P_{ij} (y-axis) information. The observed linear relationship and the perfectly matched tanglegram, showed in the right panel, highlight the strong correlation between these two metrics.

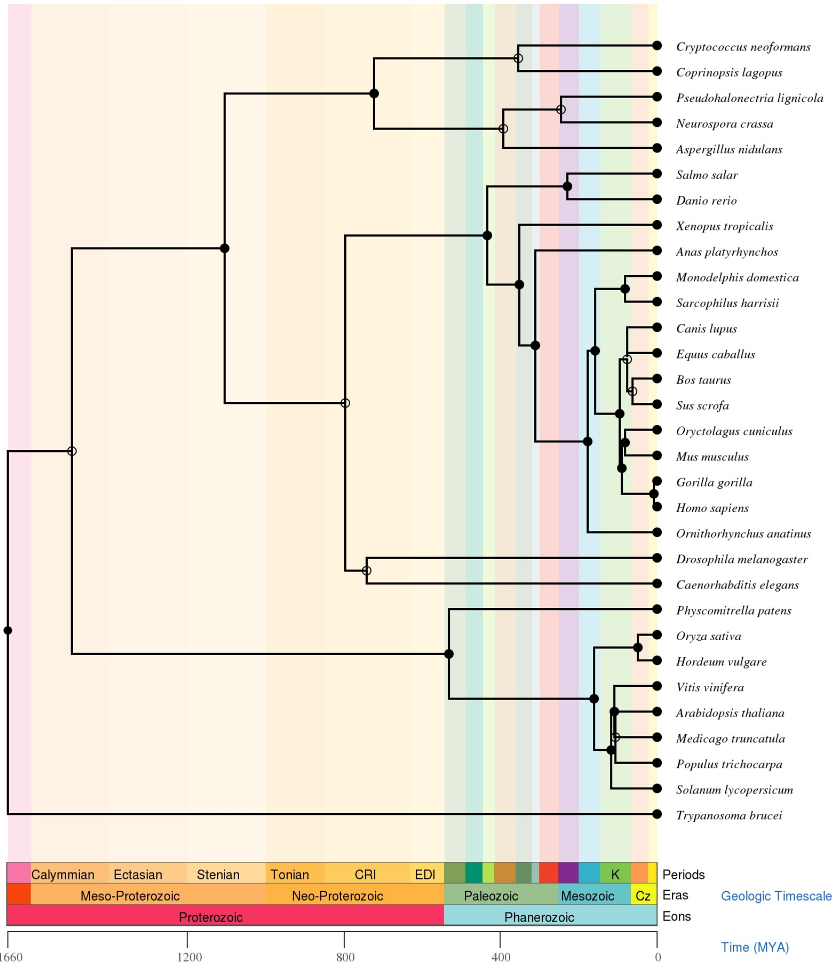

Fig C **Fig C. Phylogenetic time-tree.** Time-tree inferred from phylogenetic signals for the 30 analyzed eukaryotic species. Generated using thetimetree.org (http://www.timetree.org/search/goto-timetree, December 20, 2021).

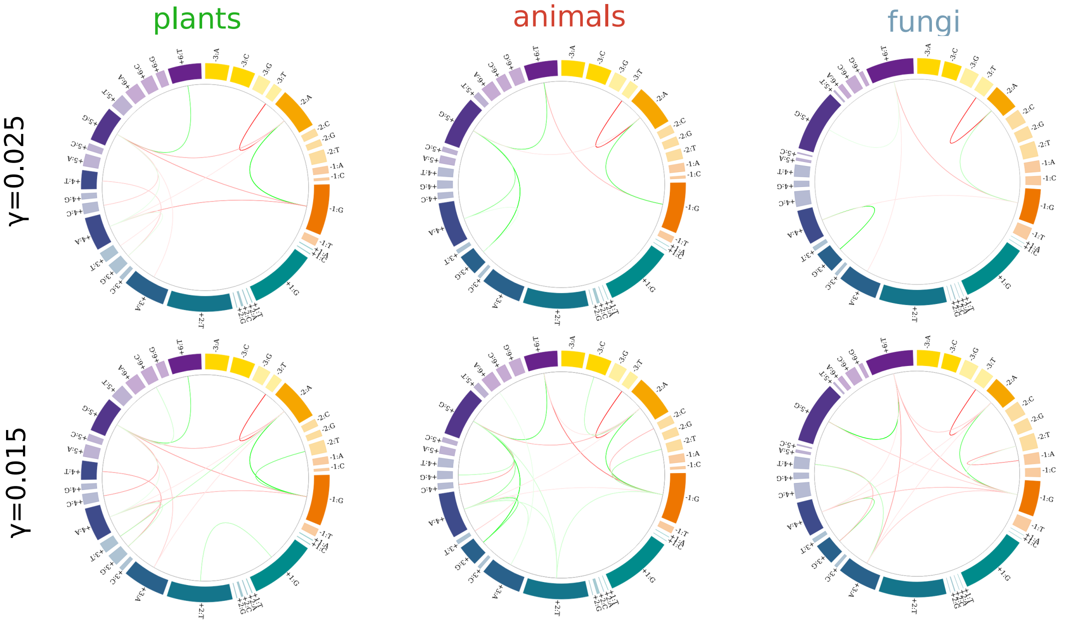

Fig D **Pairwise interaction patterns for consolidated models.** Circos diagrams for coupling patterns identified for plants, animals and fungi donor sequences are shown in the first, second and third columns respectively for $\gamma = 0.025$ and $\gamma = 0.015$ models (first and second row respectively)

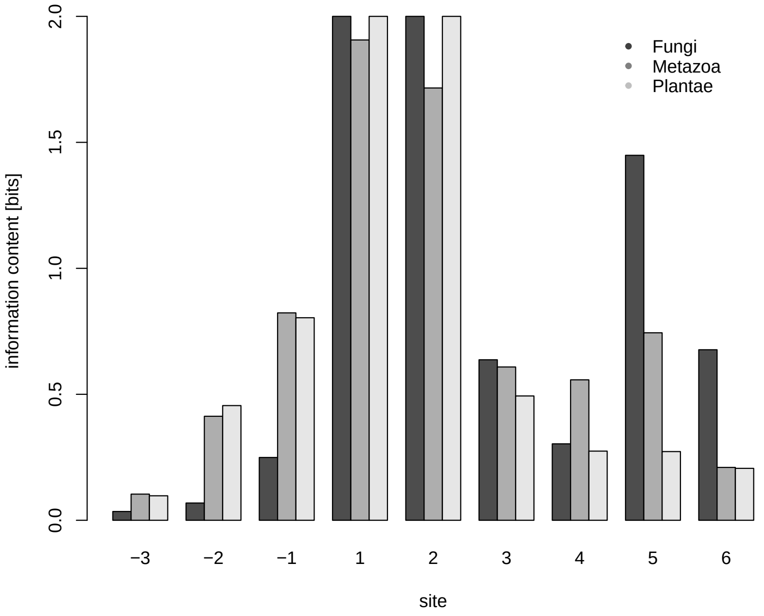

Fig E **Fig E. Information content.** Barplot of information content per site for plants, animals and fungi.

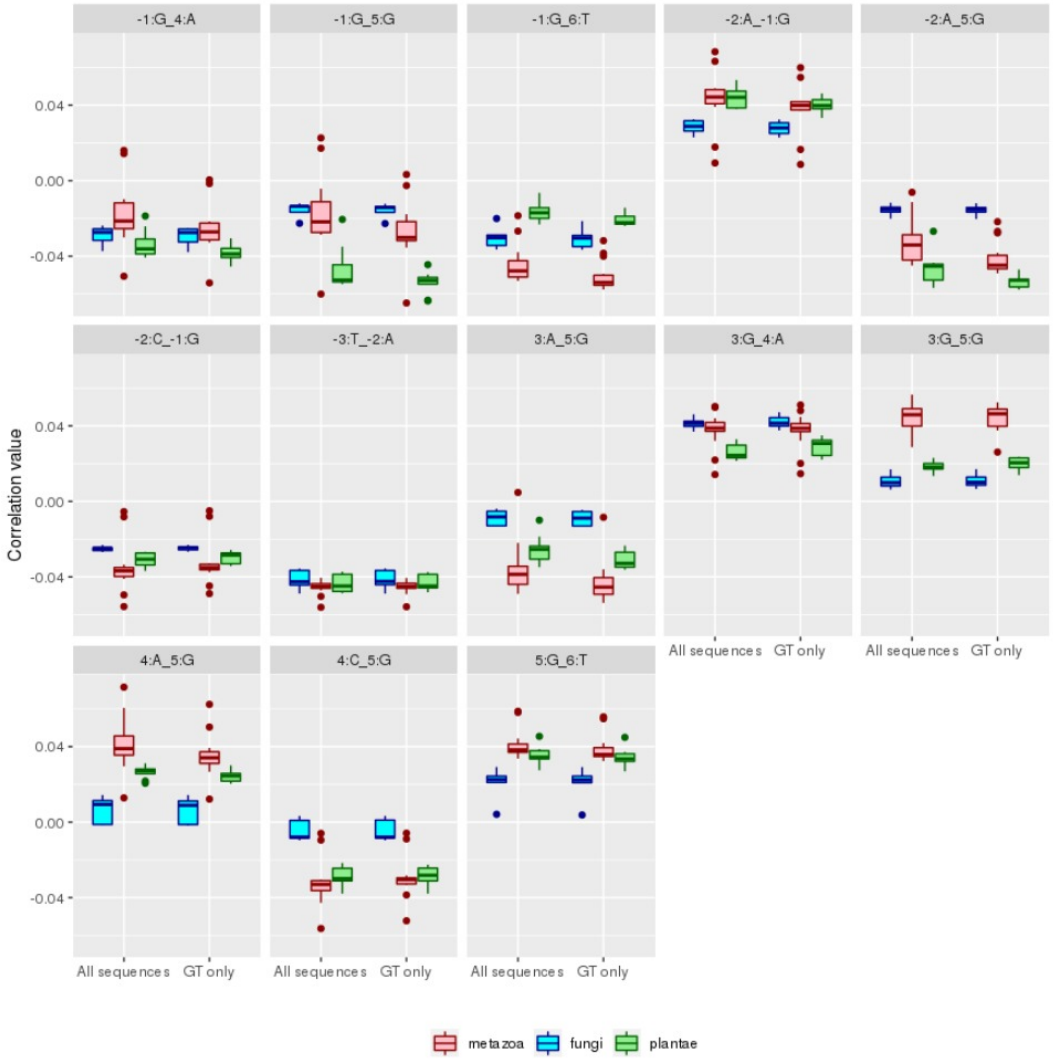

Fig F **Di-nucleotide pairwise correlations.** Boxplots summarizing the distribution of correlation values obtained for animals (red), fungi (blue) and plants (green) particular to a specific di-nucleotide pair. The first and second boxplot triplets display the complete set of annotated 5' splicing sequences and only GT 5'ss respectively.

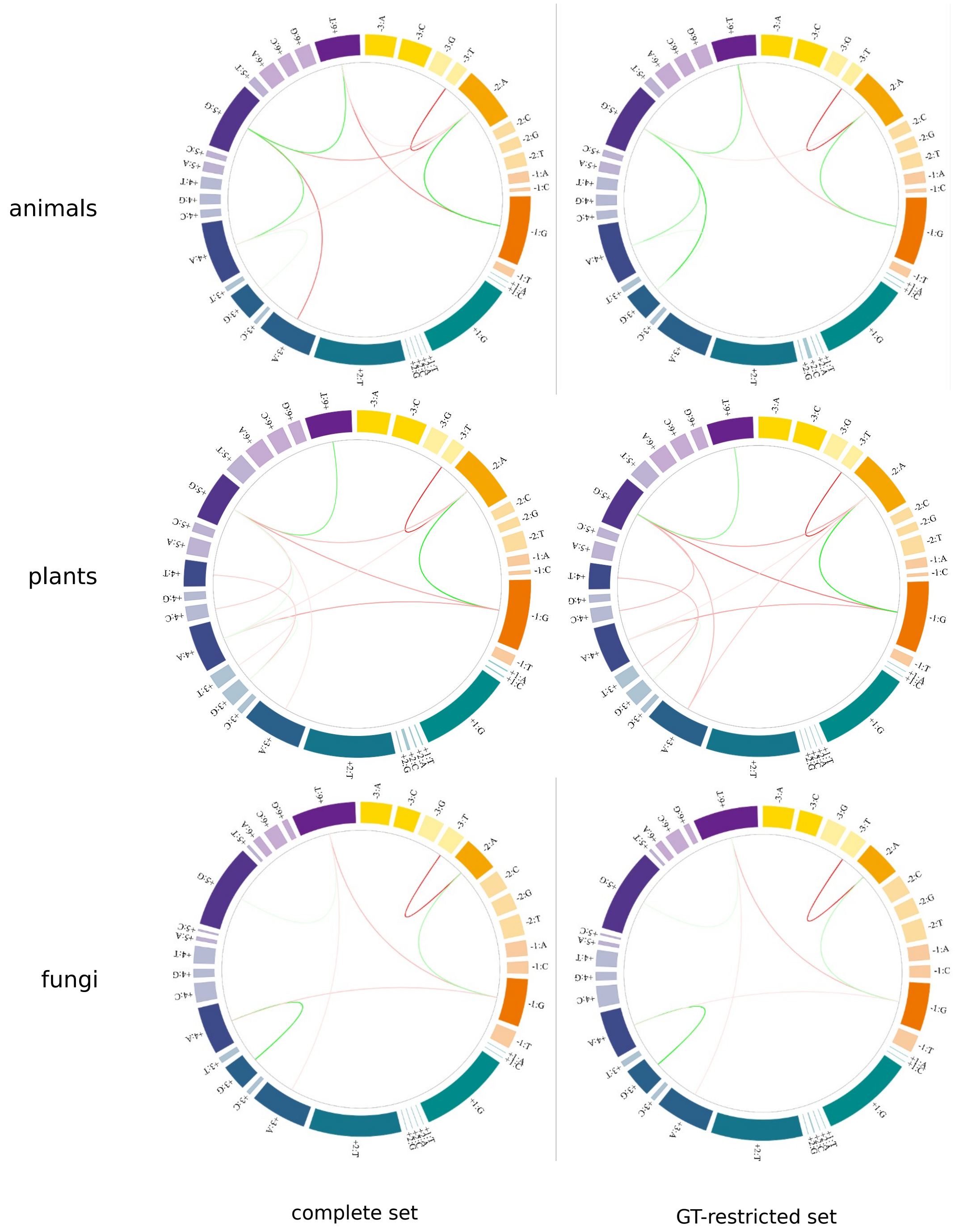

Fig G **Circos.** Circos diagrams for coupling patterns estimated for the complete set and for GT-restricted donor sequences (first and second column respectively). Results for animals, plants and fungi are shown in the first, second and third rows respectively.

References

[1] Lorenz R., Bernhart S.H., Höner Zu Siederdissen Ch. ,Tafer H. et al. (2011) ViennaRNA Package 2.0, http://www.tbi.univie.ac.at/RNA.

Supplementary Tables and Figures

- Table A: **Analyzed genomes.** For each analyzed species we show its associated assembly code, the total number of 5' exon-intron boundaries, the number of different 5'ss sequences and the number of different 5'ss sequences presenting GT as the first two intronic bases.

- Table B: **Conserved patterns ($\gamma = 0.025$).** Mean interactions between different groups of site-base occurrences, for different species. EC, ENC, IC, and INC stand for exonic-consensus, exonic-non-consensus, intronic-consensus and intronic-non-consensus occurrences respectively.

- Table C: **Conserved patterns ($\gamma = 0.015$).** Mean interactions between different groups of site-base occurrences, for different species. EC, ENC, IC, and INC stand for exonic-consensus, exonic-non-consensus, intronic-consensus and intronic-non-consensus occurrences respectively.

- Fig A: **Data driven energy.** Boxplots of data-driven energy values estimated for 502197 human 5'ss sequences for every decile of predicted dimerization energies against U1 smRNA. Dimerization free energy between the sequences of the donor sites and the complementary portion of the snRNA U1 was estimated using the program RNAcofold in the ViennaRNA 2.0 package[**?**], using default parameters.

- Fig B: **Distances inferred from one-site and two-site statistics.** The left panel shows pairwise scaled Euclidean distances between species estimated using P_i (x-axis) and P_{ij} (y-axis) information. The observed linear relationship and the perfectly matched tanglegram, showed in the right panel, highlight the strong correlation between these two metrics.

- Fig C: **Phylogenetic time-tree.** Time-tree inferred from phylogenetic signals for the 30 analyzed eukaryotic species. Generated using thetimetree.org (http://www.timetree.org/search/goto-timetree, December 20, 2021).

- Fig D: **Pairwise interaction patterns for consolidated models.** Circos diagrams for coupling patterns identified for plants, animals and fungi donor sequences are shown in the first, second and third columns respectively for $\gamma = 0.025$ and $\gamma = 0.015$ models (first and second row respectively).

- Fig E: **Information content.** Barplot of information content per site for plants, animals and fungi.

- Fig F: **Di-nucleotide pairwise correlations.** Boxplots summarizing the distribution of correlation values obtained for animals (red), fungi (blue) and plants (green) particular to a specific di-nucleotide pair. The first and second boxplot triplets display the complete set of annotated 5' splicing sequences and only GT 5'ss respectively.

- Fig G: **Circos.** Circos diagrams for coupling patterns estimated for the complete set and for GT-restricted donor sequences (first and second column respectively). Results for animals, plants and fungi are shown in the first, second and third rows respectively.
